# Opposing effects of Wnt/β-catenin signaling on epithelial and mesenchymal cell fate in the developing cochlea

**DOI:** 10.1242/dev.199091

**Published:** 2021-06-01

**Authors:** Sara E. Billings, Nina M. Myers, Lee Quiruz, Alan G. Cheng

**Affiliations:** Department of Otolaryngology – Head and Neck Surgery, Stanford University School of Medicine, Stanford, CA 94305, USA

**Keywords:** Epithelial-mesenchymal transition, β-catenin, Wnt pathway, Cochlea, Mouse

## Abstract

During embryonic development, the otic epithelium and surrounding periotic mesenchymal cells originate from distinct lineages and coordinate to form the mammalian cochlea. Epithelial sensory precursors within the cochlear duct first undergo terminal mitosis before differentiating into sensory and non-sensory cells. In parallel, periotic mesenchymal cells differentiate to shape the lateral wall, modiolus and pericochlear spaces. Previously, Wnt activation was shown to promote proliferation and differentiation of both otic epithelial and mesenchymal cells. Here, we fate-mapped Wnt-responsive epithelial and mesenchymal cells in mice and found that Wnt activation resulted in opposing cell fates. In the post-mitotic cochlear epithelium, Wnt activation via β-catenin stabilization induced clusters of proliferative cells that dedifferentiated and lost epithelial characteristics. In contrast, Wnt-activated periotic mesenchyme formed ectopic pericochlear spaces and cell clusters showing a loss of mesenchymal and gain of epithelial features. Finally, clonal analyses via multi-colored fate-mapping showed that Wnt-activated epithelial cells proliferated and formed clonal colonies, whereas Wnt-activated mesenchymal cells assembled as aggregates of mitotically quiescent cells. Together, we show that Wnt activation drives transition between epithelial and mesenchymal states in a cell type-dependent manner.

## Abstract (precis)

**Summary:** The developing cochlea comprises spatially and lineally distinct populations of epithelial and mesenchymal cells. This study shows the opposing effects of aberrant Wnt/β-catenin signaling on cell fates of cochlear epithelial and mesenchymal cells.

## INTRODUCTION

Development of the embryo requires multiple rounds of transition between epithelial and mesenchymal states, coined epithelial-mesenchymal or mesenchymal-epithelial transitions (EMT and MET, respectively) (Hay, 1995; Thiery and Sleeman, 2006). EMT is characterized by a loss of adhesion molecules and adoption of migratory behavior, whereas MET features apical-basal polarization and formation of junctional complexes, as well as the loss of cell mobility, proliferation and expression of myriad mesenchymal markers (Thiery et al., 2009). During early embryonic development, mesoderm generated by EMT gives rise to multiple epithelial organs such as the kidney and ovaries (Davies, 1996). Moreover, neural crest cells deriving from the neuroepithelium undergo EMT to become migratory mesenchymal cells, which contribute to diverse organ systems such as the cardiovascular organs, skin pigment cells, craniofacial skeleton and the peripheral nervous systems including the inner ear (Freyer et al., 2011; Martik and Bronner, 2017; Noden, 1983; Piacentino et al., 2020).

**Fig. 1.**
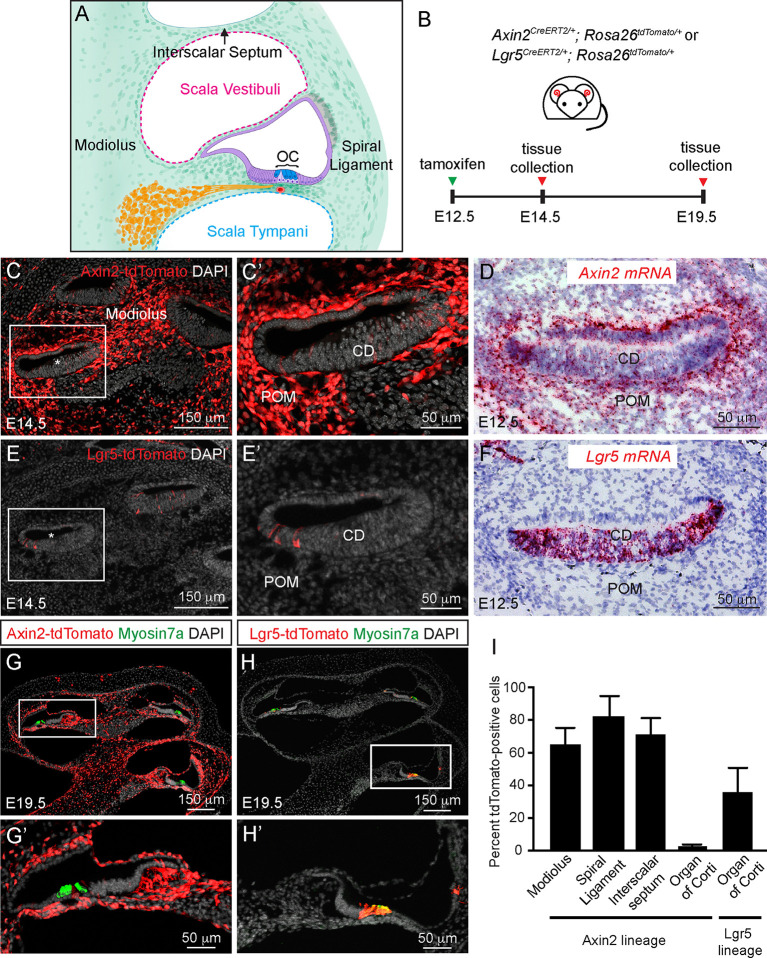
**Wnt-responsive cells in the developing cochlea.** (A) Diagram of the cochlea at E19.5. The cochlear ductal epithelium (purple) is distinguished from the structures derived from the periotic mesenchymal tissues (interscalar septum, modiolus and spiral ligament; green). Hair cells (blue) are found in the organ of Corti (OC). (B) Fate mapping was induced at E12.5 and tissues were harvested at E14.5 or E19.5. (C,C′) At E14.5, Axin2-tdTomato^+^ cells were found primarily in the modiolus and periotic mesenchyme (POM) and occasionally in the cochlear duct (CD, asterisk). (D) *Axin2* mRNA expression detected by *in situ* hybridization in the cochlear duct and periotic mesenchyme at E12.5. (E) Lgr5-traced tdTomato^+^ cells were found only in the floor of the cochlear duct (asterisk) at E14.5. (F) *Lgr5* mRNA expression was detected in the cochlear duct and not in the periotic mesenchyme at E12.5. (G-H′) At E19.5, Axin2-traced cells (G,G′) were found in mesenchymal-derived tissues including the modiolus and the spiral ligament, whereas Lgr5-traced cells (H,H′) were restricted to the epithelium. C′,E′,G′,H′ show magnifications of boxed areas in C,E,G,H, respectively. (I) Quantification of tdTomato-traced cells in Axin2-tdTomato and Lgr5-tdTomato mice. Data shown as mean±s.d. *n*=180-589 total nuclei from 3-8 different litters.

The developing mammalian cochlea consists of epithelial and mesenchymal constituents deriving from distinct lineages. The otic epithelium originates almost exclusively from the otic placode (Ohyama and Groves, 2004), harboring prosensory cells that differentiate into hair cells that are crucial for sound detection. Periotic mesenchymal cells surround the epithelium and form the modiolus, the spiral ligament in the lateral cochlear wall and also pericochlear fluid spaces (scala tympani and scala vestibuli) (Fig. 1A), which are important for the propagation of sound waves (Reichenbach and Hudspeth, 2014; Rubel and Fritzsch, 2002). Although most epithelial and mesenchymal cell types in the cochlea are considered segregated during development, several exceptions exist: precursors to spiral ganglion neurons delaminate from the otic epithelium at around embryonic day (E) 11.5 and eventually reside in the modiolus (Raft et al., 2007); neural crest cells migrate into the inner ear to give rise to glia cells in the Rosenthal's canal and pigmented intermediate cells of the stria vascularis (Freyer et al., 2011; Trowe et al., 2011).

Canonical Wnt signaling directs cell proliferation, migration and differentiation during development and regeneration (Eger et al., 2000; Logan and Nusse, 2004). Among the numerous signaling pathways implicated in regulating EMT, canonical Wnt signaling has been shown to promote mesenchymal transformation (Lamouille et al., 2014). In the developing cochlea, β-catenin, the central mediator of canonical Wnt signaling, is required for specification of the otic placode (Ohyama et al., 2006), morphogenesis of the vestibular apparatus (Riccomagno et al., 2005), differentiation and patterning of the organ of Corti (Ellis et al., 2019; Jacques et al., 2012; Jansson et al., 2019; Munnamalai and Fekete, 2016; Shi et al., 2014) and differentiation of the periotic mesenchymal cells (Bohnenpoll et al., 2014). Conversely, Wnt activation has been reported to promote proliferation and sensory cell formation in the embryonic cochlear epithelia and also proliferation in the periotic mesenchyme (Bohnenpoll et al., 2014; Jacques et al., 2012; Shi et al., 2014), suggesting a mitogenic and pro-differentiation role in both the epithelial and mesenchymal derivatives.

To directly compare the effects of Wnt activation in both the otic epithelial and mesenchymal compartments of the developing cochlea, we fate-mapped and activated Wnt signaling by stabilizing β-catenin in Wnt-responsive epithelial and mesenchymal cells. Stabilization of β-catenin increased proliferation and suppressed the expression of epithelial and differentiation markers in the otic epithelium while diminishing proliferation and expression of mesenchymal markers in the periotic mesenchyme. Both epithelial markers and ectopic formation of pericochlear spaces were found as a result of stabilization of β-catenin in the periotic mesenchyme. Clonal analysis revealed that otic epithelial cells proliferated and formed clonal colonies, whereas periotic mesenchymal cells aggregated while becoming mitotically quiescent. Together, our results reveal that Wnt activation drives a context-dependent transition along the spectrum of epithelial-mesenchymal states in the developing cochlea.

## RESULTS

### Wnt-responsive cells in the developing cochlea

As a Wnt target gene, *Axin2* marks Wnt-responsive cells in multiple organ systems (Lustig et al., 2002; van Amerongen et al., 2012a), including the cochlea (Chai et al., 2011; Jan et al., 2013). To identify Axin2*^+^* cells in the embryonic cochlea, we administered tamoxifen to E12.5 *Axin2^CreERT2/+^; Rosa26^tdTomato/+^* (Axin2-tdTomato) mice (Fig. 1B). Two days later, we found tdTomato^+^ cells primarily in the periotic mesenchyme around the cochlear duct (Fig. 1C). Occasional tdTomato^+^ cells were also detected inside the cochlear duct, including the floor epithelium where the organ of Corti later arises (Fig. 1C). Using *in situ* hybridization, *Axin2* mRNA expression was verified to be robust in the periotic mesenchyme and also to a lesser degree in the cochlear duct at E12.5 (Fig. 1D; Fig. S1A,B), corroborating Axin2-Cre activity and published results (Bohnenpoll et al., 2014; Chai et al., 2011). *Lgr5* is another Wnt target gene marking Wnt-responsive cells in developing and mature organs (Barker et al., 2010, 2007). When we administered tamoxifen to the E12.5 *Lgr5^CreERT2/+^; RosaR26^tdTomato/+^* (Lgr5-tdTomato) mice (Fig. 1B), a modest number of tdTomato^+^ cells were found exclusively inside the E14.5 cochlear ductal floor (Fig. 1E). We performed *in situ* hybridization and verified the pattern of Lgr5-Cre recombinase activity (Fig. 1F), which contrasts with that of Axin2-Cre and also corroborates previous results (Chai et al., 2011; Shi et al., 2012).

We next used these two mouse strains to fate-map Wnt-responsive cells in the cochlear duct and periotic mesenchyme in the developing cochlea. By E19.5, the cochlea has morphed to harbor several enlarged pericochlear spaces: nascent hair cells marked by myosin 7a (Myo7a) occupy the organ of Corti in the scala media, which is separated from the adjacent scala tympani and scala vestibuli by the basilar and Reissner's membranes, respectively (Fig. 1A). The interscalar septum borders the scala tympani and vestibuli. When Axin2^+^ cells were fate-mapped from E12.5 to E19.5, Axin2-tracing robustly labeled the modiolus, the spiral ligament in the lateral cochlear wall, the basilar membrane, Reissner's membrane, spiral limbus and the interscalar septum (Fig. 1G). We quantified the percentage of traced cells in three of these regions and found that most cells were Axin2-tdTomato^+^ (modiolus: 64.9±10.3%; spiral ligament: 82.11±12.6%; interscalar septum: 71.1±10.0%; mean±s.d.; Fig. 1G,I), suggesting that they derived from Axin2^+^ periotic mesenchymal cells. On the other hand, cells in the organ of Corti were rarely labeled (2.5±1.2%; Fig. 1G,I), as was expected from the low Axin2 expression in the organ of Corti at E12.5 (Fig. 1C). As expected, when Lgr5^+^ cells in the cochlear duct were tracked from E12.5 to E19.5, many were found in the organ of Corti (39.7±13.2%; Fig. 1H-I) and none found outside the scala media compartment. A small percentage of cells medial and lateral to the organ of Corti were also labeled by Lgr5 tracing (2.2±1.9% greater epithelial ridge, 17.1±1.6% lesser epithelial ridge and 17.7±1.2% of lateral wall), whereas no stria vascularis or Reissner's membrane cells were labeled (*n*=68-180 cells from three cochleae). Corn oil controls resulted in rare tdTomato^+^ cells in the Axin2-tdTomato (<1%) and none in the Lgr5-tdTomato cochleae, suggesting that Cre recombinase leakiness is highly unlikely. Together, these results demonstrate that cochlear duct epithelial cells give rise to the organ of Corti and other cells inside the scala media compartment, whereas periotic mesenchymal cells give rise to multiple components outside of the scala media compartment.

### Wnt activation via β-catenin stabilization

β-Catenin is the central mediator of Wnt canonical signaling. Phosphorylation and subsequent degradation of β-catenin render it and the pathway inactive (Ikeda et al., 1998; Yost et al., 1996). To assess the effects of active Wnt signaling on Wnt-responsive cells, we examined the Ctnnb1-flox(exon 3) mouse, in which Cre recombinase-mediated excision of exon 3 stabilizes β-catenin, leading to a constitutively active Wnt pathway (Harada et al., 1999).

**Fig. 2.**
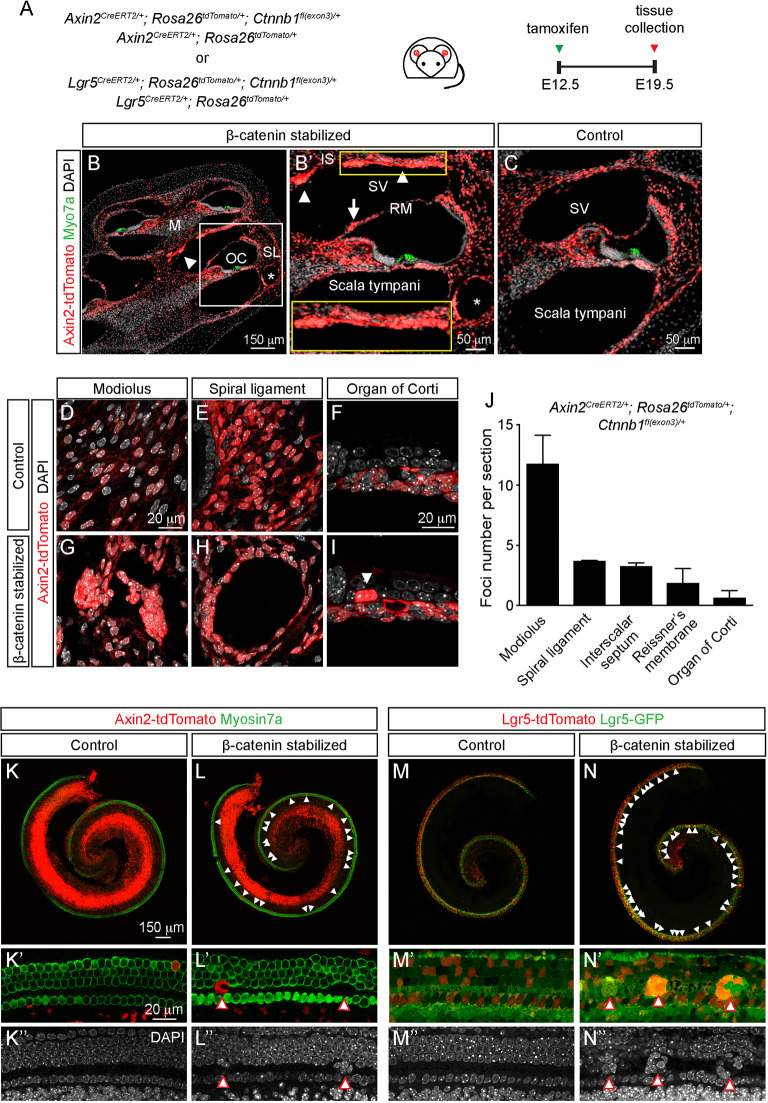
**Formation of foci in the cochlear duct and periotic mesenchyme after β-catenin stabilization.** (A) β-catenin was conditionally stabilized in Axin2^+^ or Lgr5^+^ cells following tamoxifen at E12.5 and tissues were collected at E19.5. (B,B′) Low magnification image of sections of Axin2-tdTomato-Ctnnb1 cochlea showed grossly normal morphology. Foci of Axin2-tdTomato^+^ cells were found in the periotic mesenchyme, including the modiolus (M; arrowhead, B), interscalar septum (IS; arrowheads, B′), Reissner's membrane (RM; arrow, B′) and spiral ligament (SL; asterisk). In the spiral ligament in the lateral wall, ectopic pericochlear spaces (asterisk, B,B′) lined by Axin2-tdTomato^+^ cells were observed as a result of β-catenin stabilization. B′ shows magnification of boxed area in B. Yellow box at bottom of panel shows magnification of yellow boxed area at top. (C-F) In control E19.5 Axin2-tdTomato cochlea, traced cells occupied the modiolus, spiral ligament and tympanic border cells below the organ of Corti. (G-I) Axin2-tdTomato^+^ foci were found to reside in the modiolus, spiral ligament and organ of Corti in Axin2-tdTomato-Ctnnb1 cochlea. Arrowhead (I) depicts foci in organ of Corti. (J) Quantification of foci in specific regions of the embryonic cochlea. (K-K″) Whole-mount view of the organ of Corti showing four rows of hair cells and occasional tdTomato^+^ hair cells and supporting cells. (L-L″) Axin2-tdTomato-Ctnnb1 cochlea contained numerous tdTomato^+^ and tdTomato^−^ foci along the length of the cochlea (arrowheads). The foci were found primarily in the pillar cell region and disrupted the organization of the organ of Corti. (M-M″) tdTomato^+^ hair cells and supporting cells were found in the E19.5 Lgr5-tdTomato cochlea (tamoxifen administered at E12.5). (N-N″) In the Lgr5-tdTomato-Ctnnb1 cochlea, foci were noted along the cochlear length and expressed Lgr5-EGFP. Most foci were found in the pillar cell region and also consisted of tdTomato^+^ and tdTomato^−^ cells. K′,K″,L′,L″,M′,M″,N′,N″ show magnifications of K,L,M,N, respectively. Data shown as mean±s.d. *n*=3 biological replicates from three mice, 59-100 foci per animal.

We first examined the E19.5 *Axin2-tdTomato; Ctnnb1^fl(ex3)/+^* (Axin2-tdTomato-Ctnnb1) cochlea after tamoxifen administration at E12.5 as before (Fig. 2A). We noted many clusters of bright tdTomato-labeled cells in the modiolus, Reissner's membrane and interscalar septum, whereas the compartmentalization of scala tympani and vestibuli appeared mostly preserved (Fig. 2B). Similar to cochlea from control *Axin2-tdTomato* mice, Axin2-traced cells were found in the cells surrounding the scala tympani and vestibuli in the Axin2-tdTomato-Ctnnb1 cochlea (Fig. 2B-C). Moreover, we found ectopic pericochlear spaces with clusters of Axin2-traced cells in the spiral ligament (Fig. 2B). These spaces were found throughout the length of the cochlea, but were most prominent in the base and typically located adjacent to the scala tympani.

The Axin2-tdTomato-Ctnnb1 cochleae displayed many cell clusters (termed foci) consisting of three or more densely packed cells, most of which were tdTomato-labeled (Fig. 2D-J). Unlike control cochleae, in which no cell clusters were found, many clusters occupied the Reissner's membrane, interscalar septum, modiolus and occasionally the organ of Corti (modiolus and the organ of Corti with the most and least per section, Fig. 2J). Many foci cells were tdTomato^+^ in the organ of Corti (43.6±17.8%) and the mesenchyme (89.1±3.1%), indicating that they represent progenies of Axin2-marked Wnt-responsive cells. To confirm that foci cells express stabilized β-catenin, we performed *in situ* hybridization using probes designed to detect the sequence bridging exons 2 and 4 (*Ctnnb1* ex2/4 signal), which was present only after Cre-recombinase-mediated excision of exon 3 (Fig. S2A). *Ctnnb1* ex2/4 signals were absent in control tissues (Fig. S2B,D-F) but were robust within foci in the Axin2-tdTomato-Ctnnb1 cochleae (Fig. S2C,G-I). Furthermore, immunostaining for β-catenin showed higher expression and nuclear localization of β-catenin in foci from Axin2-tdTomato-Ctnnb1 cochleae relative to other regions without foci and also relative to control tissues (Fig. S2J-L).

In the organ of Corti, Axin2-tdTomato^+^ foci primarily occupied the pillar cell region along the length of the Axin2-tdTomato-Ctnnb1 cochlea (Fig. 2K-L). These foci similarly expressed *Ctnnb1* ex2/4 mRNA signal representative of stabilized β-catenin (Fig. S2I). To verify that these foci originated from within the cochlear duct, we inspected the *Lgr5-tdTomato; Ctnnb1^fl(ex3)/+^* (Lgr5-tdTomato-Ctnnb1) cochlea, in which Lgr5-tdTomato was highly expressed in the organ of Corti and exclusively inside the cochlear duct (Fig. 1G). Similar to Axin2-tdTomato^+^ foci, Lgr5-tdTomato^+^ foci resided in the pillar cell region along the cochlea, indicating that these foci originated from Wnt-responsive cells, which expressed both *Lgr5* and *Axin2*, within the cochlear duct (Fig. 2M-N). These foci uniformly expressed Lgr5-EGFP, indicating active Wnt signaling. Together, these results show that Wnt-responsive cells formed foci of cells as a result of activating Wnt signaling via β-catenin stabilization in both the otic epithelium and the periotic mesenchyme.

### Context-dependent effects of β-catenin stabilization on proliferation

**Fig. 3.**
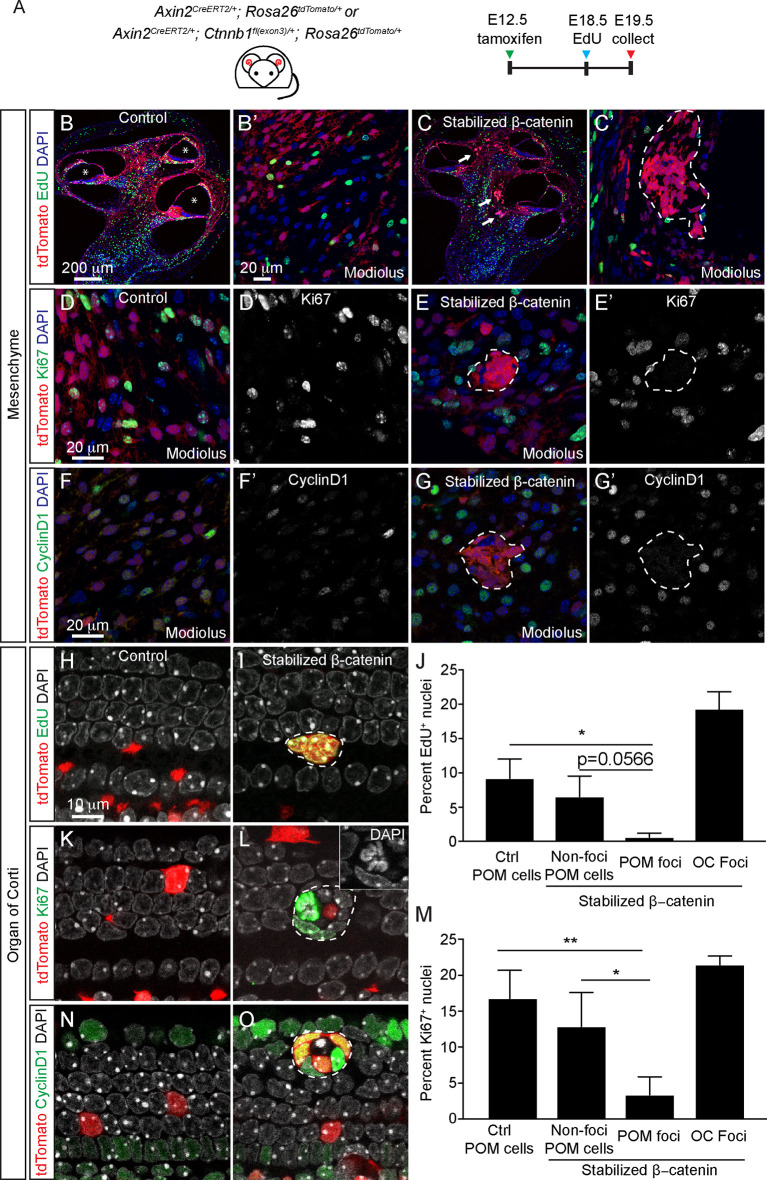
**β-Catenin stabilization increases proliferation in epithelial but not mesenchymal cells.** (A) Axin2-tdTomato-Ctnnb1 mice received tamoxifen administration at E12.5 and tissues were collected at E19.5. EdU was administered at E18.5 to label proliferative cells and Axin2-tdTomato cochleae served as controls. (B,B′) Cross-sections of control cochleae show EdU-labeled tdTomato^+^ cells primarily in the mesenchyme, including the modiolus. Asterisks mark the scala media. (C,C′) In the Axin2-tdTomato-Ctnnb1 cochlea, tdTomato^+^ foci (arrows) were found in the modiolus. They were notably EdU^−^. (D-E′) In the control, Axin2-tdTomato cochlea, many Ki67^+^, tdTomato^+^ cells were found in the modiolus (D). By contrast, tdTomato^+^ foci found in the Axin2-tdTomato-Ctnnb1 cochlea were mostly Ki67^−^ (E). (F-G′) Control Axin2-tdTomato cochlea displayed many cyclin D1^+^, tdTomato^+^ cells in the modiolus (F). tdTomato^+^ foci in the Axin2-tdTomato-Ctnnb1 cochlea were cyclin D1^−^ (G). (H,I) In the organ of Corti (OC) of Axin2-tdTomato cochlea, tdTomato^+^ cells are EdU^−^ (H). Foci found in the organ of Corti from the Axin2-tdTomato-Ctnnb1 mice (I) were EdU-labeled. (J) Quantification showing significant reduction of EdU^+^ periotic mesenchymal (POM) foci as a result of β-catenin stabilization. Within the Axin2-tdTomato-Ctnnb1 cochlea, foci cells showed a trend towards fewer EdU-labeled cells than other tdTomato^+^ non-foci POM cells. (K,L) Normally absent in the organ of Corti (control, K), Ki67 was expressed in foci in the Axin2-tdTomato-Ctnnb1 cochlea (L). (M) Quantification of Ki67^+^ nuclei showing a significant reduction in POM foci as a result of β-catenin stabilization. (N,O) Normally expressed at low levels in inner pillar cells and Hensen's cells (N), cyclinD1 was found to be highly expressed in organ of Corti foci in Axin2-tdTomato-Ctnnb1 mice (O). Data shown as mean±s.d. No differences were observed among foci from each turn. *n*=154-931 nuclei per litter from 3-4 litters. *P<0.05, **P<0.01 (one-way ANOVA with Tukey's multiple comparisons test). White dashed outline indicates foci boundaries.

Previously, Wnt agonists and β-catenin stabilization were found to enhance cell proliferation and ectopic hair cell formation in the sensory epithelium (Chai et al., 2012; Jacques et al., 2012; Shi et al., 2013) and proliferation in the periotic mesenchyme in the developing cochlea (Bohnenpoll et al., 2014). To further interrogate this, we first examined the effects of β-catenin stabilization on periotic mesenchymal cells by assessing Axin2-traced foci outside the cochlear duct from E19.5 Axin2-tdTomato Ctnnb1 cochlea. To detect proliferative cells, we injected EdU at E18.5 or immunostained for Ki67 (Mki67) at E19.5 (Fig. 3A). In the control mesenchyme, many EdU^+^ or Ki67^+^ Axin2-tdTomato cells were noted throughout the organ (Fig. 3A,B,D). By contrast, and unexpectedly, we observed a significant reduction in EdU^+^ or Ki67^+^ Axin2-traced foci cells in the Axin2-tdTomato-Ctnnb1 mesenchyme (Fig. 3C,E,J). We also immunostained for the cell cycle marker cyclin D1 and found its expression similarly reduced in Axin2-traced foci cells with β-catenin stabilization (Fig. 3F-G).

In contrast, quantification of EdU or Ki67 labeling in the control Axin2-tdTomato organ of Corti revealed no positive cells (*n*=418 and 72 cells from three cochlea, respectively), consistent with previous reports of mitotic quiescence by E19.5 (Fig. 3H,K) (Ruben, 1967). Conversely, β-catenin stabilization in the Axin2-tdTomato-Ctnnb1 cochleae resulted in significantly more EdU- and Ki67-labeled Axin2-traced foci cells (Fig. 3I,J,L,M). Moreover, Axin2-traced foci cells in the organ of Corti expressed cyclin D1 (Fig. 3N,O). Together, these data suggest that β-catenin stabilization increased proliferation of foci in the cochlear duct while decreasing that of foci formed by the periotic mesenchymal cells.

### Clonal analysis of Wnt-activated foci

**Fig. 4.**
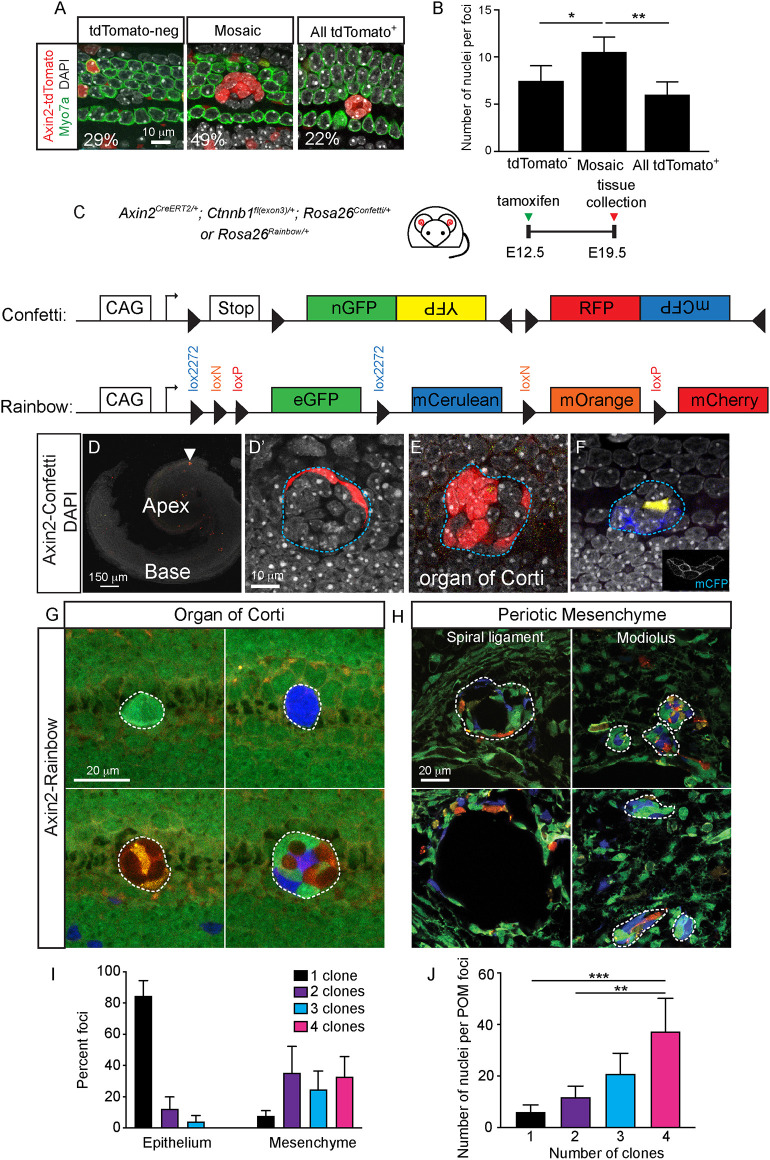
**Clonal analysis of epithelial and mesenchymal foci.** (A) In Axin2-tdTomato-Ctnnb1 cochlea, three types of foci were observed in the organ of Corti: 29% were tdTomato^−^, 49% mosaically-labeled, and 22% completely tdTomato^+^. (B) Mosaic foci were significantly larger than either tdTomato^−^ or completely tdTomato^+^ foci. *n*=97 foci from 12 mice. (C) Axin2-Ctnnb1 mice were crossed with two multicolor Cre reporter mouse lines to assess clonality of foci formed as a result of β-catenin stabilization (Rosa26R-Confetti, Rosa26R-Rainbow). (D) In the Axin2-Confetti-Ctnnb1 cochlea, there was sparse labeling of foci (arrowhead), although the number of foci per cochlea was comparable with Axin2-tdTomato-Ctnnb1 cochlea. (D′,E) High magnification images showing mosaic RFP-labeled foci. (F) High magnification image of a multi-colored foci containing clones of multiple cells of the same color (YFP and mCGP). Blue dashed lines indicate foci boundaries. (G) In the organ of Corti in Axin2-Rainbow-Ctnnb1 cochlea, more than 80% of labeled foci were single-colored (eGFP, mCerulean, mOrange or mCherry) with a small proportion being multicolored. Multiple cells of the same color adjoined each other, suggesting that they were clonally related. (H) In the periotic mesenchyme of Axin2-Rainbow-Ctnnb1 cochlea, most foci were multicolored. White dashed lines indicate foci boundaries. (I) Distribution of the number of clones per foci in the organ of Corti and periotic mesenchyme from the Axin2-Rainbow-Ctnnb1 cochlea. *P*<0.0001 (Chi-squared test). (J) Counts of nuclei per foci in the periotic mesenchyme from the Axin2-Rainbow-Ctnnb1 cochlea. Data shown as mean±s.d. *n*=1415 nuclei in 61 foci from four mice. *P<0.05, **P<0.01, ***P<0.001 [one-way ANOVA with Tukey's multiple comparisons test (B,J) or Chi-squared test (I)].

Because of the opposing effects on proliferation by β-catenin stabilization in foci in the cochlear duct and periotic mesenchyme, we hypothesize that the former was assembled via clonal expansion of individual Wnt-activated cells whereas the latter was formed via aggregation. We first examined 97 foci in 15 Axin2-tdTomato-Ctnnb1 cochleae and found that 49% were mosaic and displayed a mixture of tdTomato^+^ and tdTomato^−^ cells (Fig. 4A,B). The remaining foci were monochromatic, containing either tdTomato^+^ or tdTomato^−^ cells only (29% or 22%, respectively) (Fig. 4A,B). Quantification showed that mosaic foci contained significantly more cells than monochromatic foci (*P*<0.05; Fig. 4B). Because *in situ* hybridization showed that foci cells uniformly displayed *Ctnnb1ex2/4* transcripts (Fig. S2G-I), we hypothesize that tdTomato^+^ and tdTomato^−^ cells arose from clonal expansion of separate founder Wnt-responsive cells. To test this hypothesis, we employed two multi-color Cre reporter mouse strains, *Rosa26R-Confetti* and *Rosa26R-Rainbow* (Rinkevich et al., 2011; Snippert et al., 2010), for clonal analysis of foci in the Axin2-Ctnnb1 cochlea (Fig. 4C).

In both the Axin2-Confetti-Ctnnb1 and Axin2-Rainbow-Ctnnb1 mice, tamoxifen administration results in permanent labeling of individual Axin2^+^ cells with one of several fluorescent markers. For the Axin2-Confetti-Ctnnb1 mice, recombined Axin2^+^ cells expressed one of the four colors (mCFP, nGFP, YFP or RFP). On the other hand, Axin2^+^ cells in Axin2-Rainbow-Ctnnb1 mice expressed eGFP in the absence of Cre recombination and switched to mCerulean, mOrange or mCherry upon recombination (Rinkevich et al., 2011). With this fate-mapping approach for Axin2-traced foci, we expect clones derived from proliferation of individual cells to be the same color and to be adjacent to one another. Conversely, foci composed of clonally unrelated cells would likely display different colors.

In Axin2-Confetti-Ctnnb1 mice, tamoxifen induced sparse labeling of a subset of organ of Corti foci (12±3% of 43 foci from two animals; Fig. 4D-F). Foci contained fluorescently-labeled cells adjacent to one another, suggesting that these Axin2-traced cells were clonally related. We postulate that foci with two groups of fluorescently-labeled cells had likely arisen from two separate Axin2^+^ founder cells. To verify this possibility, we analyzed the Axin2-Rainbow-Ctnnb1 cochleae, in which many foci displayed Cre recombination (25.2%). Using this approach, we found that ∼80% of organ of Corti foci contained one group of fluorescently-labeled cells (hence single clones), and the presence of multiple clones was less common (*n*=158 foci; Fig. 4G,I,J). Together these results further support β-catenin stabilization leading to proliferation and clonal expansion of Wnt-responsive cells in the cochlear duct.

In the periotic mesenchyme of Axin2-Rainbow-Ctnnb1 cochlea, foci cells in the modiolus, interscalar septum and spiral ligament displayed multiple fluorescent labels (Fig. 4H; Fig. S3A,B), indicating the presence of multiple clones. Multicolored foci also occupied the extranumerary pericochlear spaces adjacent to the scala tympani (Fig. 4H). Unlike foci in the organ of Corti, Axin2-traced cells (expressing mCerulean, mOrange or mCherry) were often adjacent to cells of a different color and did not form foci with adjacent identically labeled cells. Overall, mesenchymal foci contained significantly more clones than organ of Corti foci [χ^2^ (2, *n*=202)=116.2, *P*<0.0001]. These results indicate that periotic mesenchymal foci formed as a result of β-catenin stabilization are multiclonal. Considering that they also showed a reduced level of proliferation (Fig. 3I,L,O), these foci likely formed as a result of aggregation of individual Axin2^+^ cells, as opposed to the clonal expansion resulting from the active proliferation in the cochlear duct.

### Wnt activation induced dedifferentiation of cochlear ductal cells

**Fig. 5.**
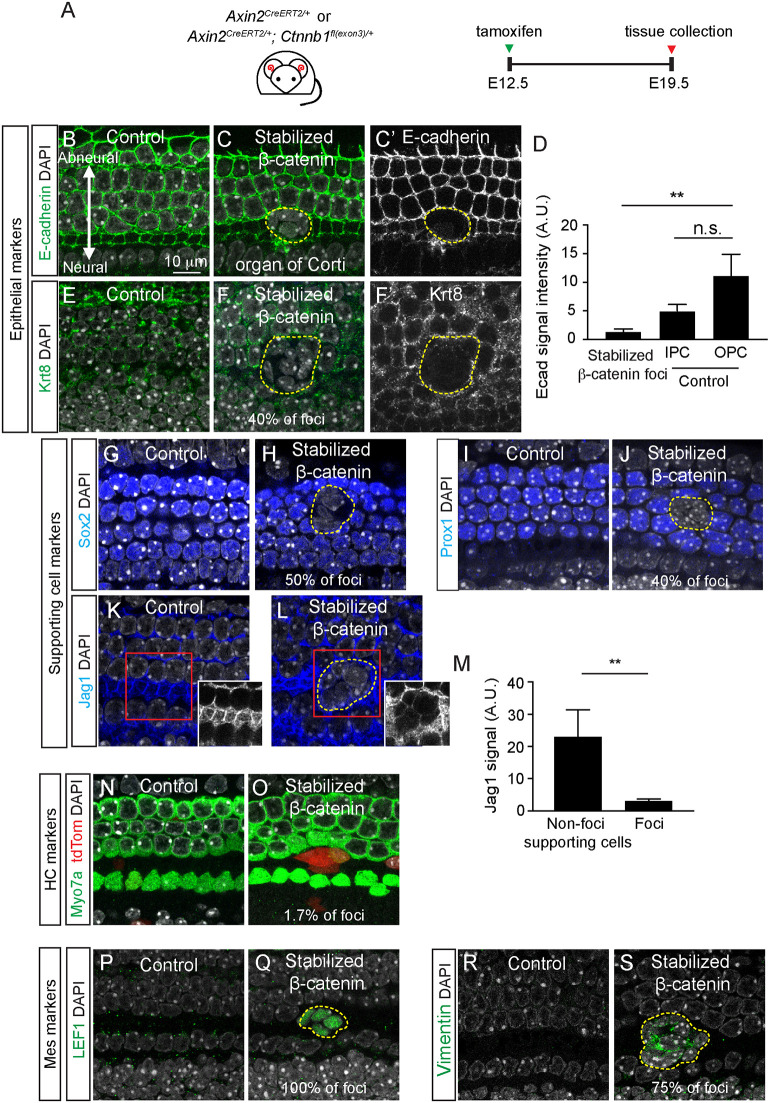
**β-Catenin stabilization causes dedifferentiation of epithelial foci.** (A) Experimental scheme to stabilize β-catenin in Axin2-Ctnnb1 cochlea. (B) In control cochlea, E-cadherin was robustly expressed in supporting cells of the organ of Corti. (C,C′) With β-catenin stabilization, foci displayed no or reduced E-cadherin expression. (D) Quantification of E-cadherin signal intensity showing a significant decrease compared with outer pillar cells (OPC) outside the foci. IPC, inner pillar cells. (E) Control cochlea with supporting cells expressing Krt8. (F,F′) Few foci in the organ of Corti from Axin2-Ctnnb1 cochlea showed Krt8 expression (∼40% foci). (G-M) Expression of the supporting cell markers Sox2 (G,H), Prox1 (I,J) and Jag1 (K-M) all decreased in foci compared with controls. (N,O) The hair cell marker Myo7a was rarely (∼1.7%) found in β-catenin-stabilized foci. (P,Q) Normally expressed in the periotic mesenchyme and absent in the organ of Corti, LEF1 was found expressed in every foci examined in the Axin2-Ctnnb1 cochlea. (R,S) Absent in the control organ of Corti and expressed in the periotic mesenchyme, vimentin is upregulated in most (∼75%) organ of Corti foci. Data shown as mean±s.d. *n*=3 cochlea from three mice from three litters. For E-cadherin (D) and Jag 1 (M), *n*=2-7 foci from three litters. **P<0.01 [one-way ANOVA with Tukey's multiple comparisons test (D) and Student's *t*-test (M)]. Dashed lines indicate foci boundaries.

Cells lining the cochlear duct are known to express epithelial markers, including E-cadherin and EpCAM (Hertzano et al., 2011; Simonneau et al., 2003). We confirmed that supporting cells in the organ of Corti express these markers and keratin 8 (Krt8) (Fig. 5A,B,E; Fig. S4C). In Axin2-tdTomato-Ctnnb1 mice, foci formed as a result of β-catenin stabilization displayed reduced expression of all three epithelial markers, whereas expression of tight junction marker ZO1 (Tjp1) was maintained (Fig. 5A-F; Fig. S4A-D). When the intensity of E-cadherin expression was quantified, it was significantly lower in foci cells than in non-foci supporting cells (outer pillar cells; Fig. 5D).

Wnt activation has previously been shown to induce supporting cell proliferation and ectopic hair cell formation in the embryonic and neonatal cochleae (Chai et al., 2012; Jacques et al., 2012; Shi et al., 2013). As non-sensory cells, supporting cells (Deiters’ and pillar cells) are marked by Sox2, Prox1 and Jag1 (Bermingham-McDonogh et al., 2006; Kiernan et al., 2005; Morrison et al., 1999). In the Axin2-tdTomato-Ctnnb1 cochleae, many foci located in the organ of Corti failed to express Sox2, Prox1 or Jag1 (Fig. 5G-M). Similarly, markers of hair cells (Atoh1 and Myo7a) were not detected in most (>98%) foci cells (Fig. 5N,O; Fig. S4E,F). Analysis of the fate of foci cells at later ages was precluded by tamoxifen-induced dystocia and perinatal lethality of the mutant animals. These results suggest that β-catenin stabilization causes dedifferentiation of cochlear epithelial cells.

Cochlear periotic mesenchymal cells express mesenchymal markers such as vimentin, Sox9, Zeb1, LEF1 and Pou3f4 (Ahn et al., 2009; Hertzano et al., 2011). With the exception of Sox9, these markers are notably absent in the cochlear duct of control Axin2-tdTomato mice (Fig. 5P,R; Fig. S4G,I). By contrast, in the organ of Corti foci, we found robust expression of LEF1, vimentin, and Sox9, but not Pou3f4 or Zeb1 (Fig. 5Q,S; Fig. S4H,J).

To verify these changes in the cochlear duct, we examined Lgr5-tdTomato-Ctnnb1 cochleae (Fig. S5A), in which Lgr5-traced cells are exclusively derived from epithelial and not mesenchymal cells (Fig. 1E,H). In Lgr5-tdTomato-Ctnnb1 foci, we similarly found a marked decrease in epithelial markers (E-cadherin, Krt8, Sox2) and an increase in proliferation (Ki67) and mesenchymal markers (vimentin and LEF1) (Fig. S5B-I), confirming that the observed phenotype is a result of β-catenin stabilization in epithelial cells.

In summary, our data suggest that β-catenin stabilization causes proliferation, dedifferentiation and a transition from an epithelial towards a mesenchymal cell state in the otic epithelium.

### Wnt activation upregulated epithelial markers of periotic mesenchymal cells

**Fig. 6.**
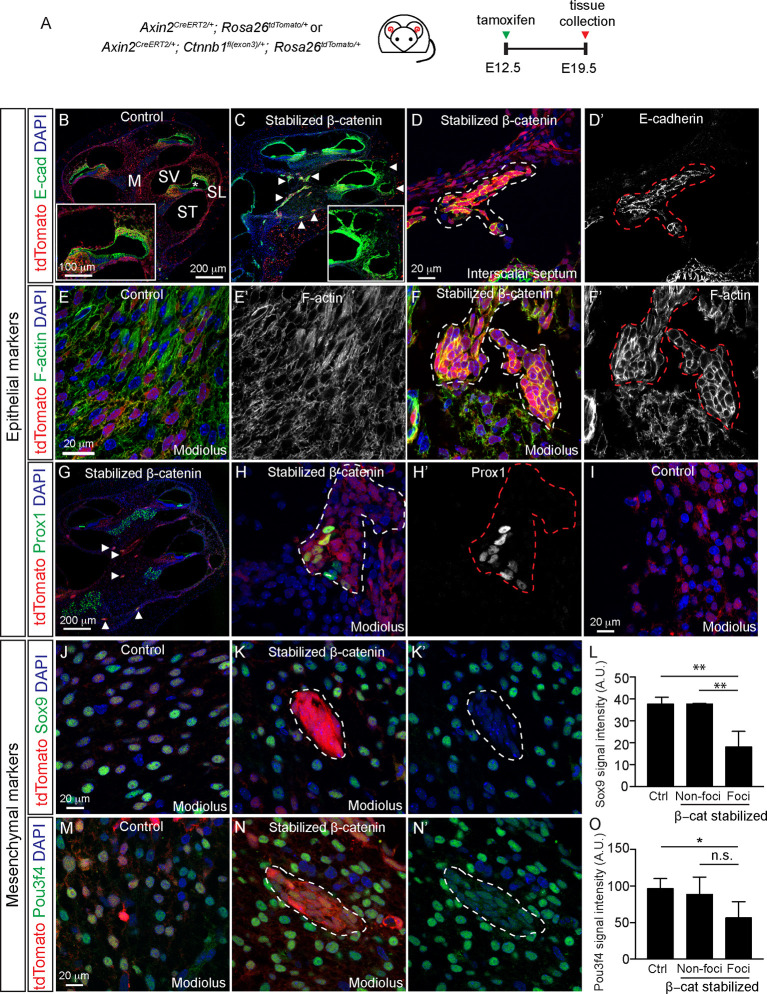
**β-Catenin stabilization induces fate switch of mesenchymal cells.** (A) Experimental scheme to fate-map and stabilize β-catenin in Axin2^+^ cells in the embryonic cochlea. (B) E-cadherin expression is restricted to the cochlear ductal epithelium (asterisk) in the control animals. Inset shows magnified basal turn of the cochlea. (C) In the Axin2-tdTomato-Ctnnb1 cochlea, ectopic expression of E-cadherin (arrowheads) was found in the modiolar (M) region and also around the lining of the ectopic pericochlear spaces in the spiral ligament (inset). (D,D′) Ectopic E-cadherin expression was restricted to the morphologically abnormal foci (dashed line). Shown image taken from the interscalar septum. (E-F′) Uniform and diffuse Phalloidin-labeled cells were seen in the control modiolus (E,E′), but distinct and banded Phalloidin-labeled foci cells were seen in the modiolus of Axin2-tdTomato-mice (F,F′). (G-I) Prox1 is ectopically expressed in the foci of β-catenin stabilized cochlea (arrowheads) (G-H′), whereas it is absent in all mesenchymal tissues in the control (I). (J) Sox9 expression in mesenchymal cells in the modiolus from control cochlea. (K,K′) Sox9 expression was downregulated in foci cells from β-catenin stabilized cochlea. (L) Quantification of the fluorescence intensity of Sox9 showing a significant reduction in β-catenin stabilized foci. (M) Pou3f4 expression in mesenchymal cells in the modiolus of control cochlea. (N,N′) Pou3f4 expression appeared diminished in foci of β-catenin stabilized cochlea. (O) Quantification of the fluorescence intensity of Pou3f4 showing a significant reduction in foci cells. Three groups analyzed were the morphologically-abnormal foci from Axin2-tdTomato-Ctnnb1 mice (Foci; β-cat stabilized), morphologically normal non-foci cells in Axin2-tdTomato-Ctnnb1 mice (Non-foci; β-cat stabilized) and cells from the same region (i.e. modiolus) in control Axin2-tdTomato mice (Ctrl). SL, spiral ligament; ST, scala tympani; SV, scala vestibuli. Data shown as mean±s.d. *n*=7-13 foci from 3-4 litters. *P<0.05, **P<0.01 (one-way ANOVA with Tukey's multiple comparisons test). n.s., not significantly different.

The epithelial markers E-cadherin and ZO1 are normally absent or expressed at low levels in the periotic mesenchymal cells (Fig. 6A,B; Fig. S6A,B). However, periotic mesenchymal foci of the Axin2-tdTomato-Ctnnb1 cochlea robustly expressed both E-cadherin and ZO1 (Fig. 6C,D and Fig. S6C). We also found E-cadherin expression lining the ectopic pericochlear spaces (Fig. 6C), suggesting the formation of an epithelial lining. In control cochlea, bands of filamentous actin appeared thin and disorganized between periotic mesenchymal cells (Fig. 6E). After β-catenin stabilization, foci showed thickened bands of filamentous actin, a finding that has been reported in sensory epithelia with diminished proliferative capacity (Fig. 6F) (Burns et al., 2008; Burns and Corwin, 2014). Foci also occasionally expressed the supporting cell markers Sox2 and Prox1, which were absent in the periotic mesenchyme of control animals (Fig. 6G,H,I; Fig. S6J), However, there was no expression of EpCAM or Krt8 (Fig. S6G,I), suggesting that β-catenin induced mesenchymal cells to acquire some but not all features of epithelial cells. To explore the possibility of MET, we immunostained for Sox9, Pou3f4 and Zeb1, which are expressed in control periotic mesenchymal cells (Fig. 6J,M; Fig. S6D). In periotic mesenchymal foci formed as result of β-catenin stabilization, we found significantly decreased expression of Sox9 and Pou3f4, but not Zeb1 (Fig. 6J-L,N-O; Fig. S6E; Table 1). In summary, β-catenin stabilization decreased proliferation of the periotic mesenchymal cells, causing them to aggregate and undergo a partial MET.

**Table 1.**
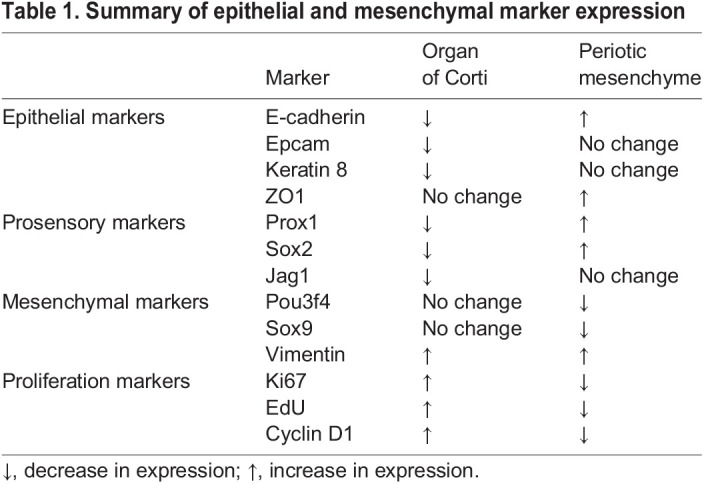
Summary of epithelial and mesenchymal marker expression

## DISCUSSION

Development of the mammalian cochlea requires the precise and coordinated morphogenesis and differentiation of epithelial and mesenchymal cell types. The cochlear epithelium deriving from the otic placode contains prosensory cells and gives rise to sensory and non-sensory cells in the organ of Corti (Driver et al., 2013; Ohyama and Groves, 2004). Periotic mesenchymal cells morph into pericochlear spaces crucial for hearing (Rubel and Fritzsch, 2002), but also govern cochlear length and sensory cell specification via FGF signaling (Huh et al., 2015). In addition, epithelial cells delaminate and populate the Rosenthal's canal as neurons, and both epithelial and mesenchymal derivatives contribute to the development of the stria vascularis in the lateral cochlear wall (Breuskin et al., 2010; Raft et al., 2007; Trowe et al., 2011). Previous work has demonstrated that Wnt/β-catenin signaling is required for differentiation of the otic placode, morphogenesis of the vestibular apparatus, and patterning and differentiation of the cochlear epithelium and periotic mesenchyme (Bohnenpoll et al., 2014; Jacques et al., 2012; Jansson et al., 2019; Landin Malt et al., 2020; Najarro et al., 2020; Ohyama et al., 2006; Riccomagno et al., 2005; Shi et al., 2014). Although Wnt-responsive cells reside in both the cochlear epithelium and periotic mesenchyme (Chai et al., 2011), whether they respond similarly to Wnt activation is unknown.

In this study, we fate-mapped Wnt-responsive epithelial and mesenchymal cells in the developing cochlea and found that Wnt activation via β-catenin stabilization led to opposing cell fates. In the cochlear epithelium, Wnt activation led to formation of foci containing dedifferentiated and proliferative cells with some mesenchymal features, suggestive of an EMT. By contrast, ectopic Wnt activation in the periotic mesenchyme induced formation of ectopic pericochlear spaces and foci with diminished proliferation and mesenchymal characteristics, implicating an MET (Table 1). Together these results underscore the complex and context-dependent effects of Wnt/β-catenin signaling.

### Multiple roles of Wnt/β-catenin signaling

As the central mediator of the canonical Wnt pathway, β-catenin carries out its Wnt pathway function via co-activation of target genes in the nucleus, though it also plays an important role in cell-cell junctions as a component of adherens junctions (Heuberger and Birchmeier, 2010).

Wnt signaling plays myriad roles in the developing cochlea (Jansson et al., 2015). In the embryonic (E12.5) cochlea, Wnt activation via chemical inhibition of GSK3β and β-catenin stabilization has been shown to broaden the prosensory domain and enhance proliferation within it (Jacques et al., 2012; Samarajeewa et al., 2018; Shi et al., 2014). In agreement with our results, a reduction of E-cadherin expression was noted after Wnt activation, which can be attributed to the role of β-catenin in cell adhesion. In contrast to these studies, we observed a downregulation or loss of markers of epithelial cells or supporting cells after β-catenin stabilization and expression of some mesenchymal markers, suggestive of dedifferentiation and EMT.

One should note that our study has employed an approach in which a limited number of Wnt-responsive cells were subjected to Wnt activation, thereby facilitating clonal analysis of the cell-autonomous effects of stabilized β-catenin. It is possible that when Wnt activation concurrently occurs among numerous cochlear epithelial/supporting cells, Wnt-activated cells may recruit adjacent cells in a non-cell autonomous fashion. Such a phenomenon has been observed in the hair follicle, where stem cells with stabilized β-catenin recruit adjacent stem cells via Wnt secretion (Deschene et al., 2014). Lastly, it is likely that different levels of Wnt activation exert differential effects on cell fate, and that an intermediate level of Wnt/β-catenin signaling guides proper development of both cochlear epithelial and mesenchymal cells.

We do not exclude the possible effects of Axin2 haploinsufficiency on the results presented. As a negative feedback inhibitor of the Wnt/β-catenin signaling, Axin2^+^ cells from *Axin2-CreERT2* mice have been shown to respond more robustly to Wnt activation (Zeng and Nusse, 2010). Although this possibility is small, as cochlear epithelial cells from *Lgr5-CreERT2* mice also formed foci and phenocopied those from *Axin2-CreERT2* mice, we do not rule out such a confounding effect. Such an interactive effect has been seen with Sox2 haploinsufficiency and Wnt activation in the postnatal cochlea (Atkinson et al., 2018).

As stated above, β-catenin both acts as a transcriptional activator of Wnt signaling and regulates cell adhesion. Based on the formation of ectopic pericochlear spaces and aggregates displaying E-cadherin and actin bands, we postulate that the junctional properties of β-catenin contribute at least in part to decrease proliferation and MET. The increase in E-cadherin expression may also contribute to the formation of multi-clonal foci in the mesenchyme, as differential cadherin expression allows self-sorting of cells *in vitro* (Nose et al., 1988). Recently, the cell adhesion properties of β-catenin were shown to govern radial patterning of the cochlear duct (Jansson et al., 2019). How the cell adhesion versus transcriptional effects of β-catenin coordinate to regulate the periotic mesenchyme is unknown and the current dataset should serve as the foundation to further explore this open question.

### Epithelial-mesenchymal transition

The developing cochlea is composed of distinct epithelial and mesenchymal cell lineages. Our results show that the epithelial and mesenchymal cell characteristics may be altered after aberrant activation of Wnt/β-catenin signaling. In support of this cochlear cell plasticity, EMT has been reported after ototoxic injury *in vivo* and in dissociated sensory epithelial cells *in vitro* (Ladrech et al., 2017; Zhang and Hu, 2012). Conversely, MET can also occur with misregulation of Zeb1 *in vivo* (Hertzano et al., 2011).

A body of literature shows that active Wnt/β-catenin signaling induces EMT during development and carcinogenesis (Logan and Nusse, 2004). For example, Wnt-mediated EMT has been implicated in the pathogenesis of cancer of the mammary glands, ovaries and the intestines (Reya and Clevers, 2005). In the developing kidney, the metanephric mesenchyme forms the distal tubules in a Wnt-dependent fashion (Kispert et al., 1998). Similar to what we have observed in the periotic mesenchyme in the developing cochlea, overactivation of the Wnt pathway results in ectopic epithelialization, suggesting it is both required and sufficient for MET (Kuure et al., 2007; Sarin et al., 2014; Schmidt-Ott et al., 2007). The mechanisms underlying the differences in cellular response (EMT versus MET) with Wnt activation are largely unknown.

The formation of proliferative foci with dedifferentiated cells in the cochlear epithelium as a result of Wnt activation resembles tumor growth in many other organ systems. In epidermal-derived organs such as skin and mammary glands, activation of Wnt signaling and upregulation of associated target genes are commonplace (Logan and Nusse, 2004). Surprisingly, β-catenin stabilization in the periotic mesenchyme resulted in diminished proliferation, loss of mesenchymal markers and upregulation of epithelial characteristics, implicating an MET. This conclusion was further supported by clonal analysis using multicolor Cre reporters, showing frequent mosaicism with β-catenin stabilization, likely as a result of cell aggregation. Instead of distinct epithelial and mesenchymal fates, intermediate states exist in many tissue types, suggesting a spectrum rather than distinct epithelial or mesenchymal states (Nieto et al., 2016). In our study, EMT and MET observed as a result of Wnt activation likely represent such intermediate states.

Wnt ligands have been reported to exert different cellular effects depending on types of receptors present on Wnt-responsive cells (van Amerongen et al., 2012b, 2008). Although Wnt activation is classically viewed as a mitogenic and self-renewal signal in otic and non-otic tissues (Jansson et al., 2015; Logan and Nusse, 2004), it has also been shown to suppress proliferation and tumor formation in subtypes of medulloblastoma and breast cancer (Green et al., 2013; Manoranjan et al., 2020). Likewise, our results indicate that the cellular effects of β-catenin stabilization are highly context-dependent.

In summary, our study shows that Wnt activation via β-catenin stabilization causes opposing cell fate changes in epithelial and mesenchymal cells in the developing cochlea. As it is possible that damaged/regenerating tissues contain various Wnt-responsive cell types, the context-dependent effects of Wnt activation may have broad implications in regenerative medicine.

## MATERIALS AND METHODS

### Mice

The transgenic mouse lines *Axin2^CreERT2^* (van Amerongen et al., 2012a) and *Lgr5^CreERT2^* (Barker et al., 2007) were used. For lineage tracing, *Axin2^CreERT2^* and *Lgr5^CreERT2^* mice were bred with the reporter strains *Rosa26R^tdTomato^* (The Jackson Laboratory, 7908) (Madisen et al., 2010), *Rosa26R^Confetti^* (Snippert et al., 2010) (gift of H. Clevers, Utrecht University, Netherlands) and *Rosa26R^Rainbow^* (Rinkevich et al., 2011) (MGI-5441200, gift of I. Weissman, Stanford University, USA). *Ctnnb1^fl(ex3)/+^* mice (MGI-1858008, gift of M. Taketo, Kyoto University, Japan) were crossed with the above Cre lines to conditionally stabilize β-catenin (Harada et al., 1999). Tamoxifen was delivered at E12.5 using gavage (100 µg/g) for *Axin2^CreERT2^* animals, or by intraperitoneal injection (150 µg/g) for *Lgr5^CreERT2^* animals. Three doses of EdU (10 mg/kg; Invitrogen) were administered by intraperitoneal injection at 2 h intervals on E18.5. Corn oil was used for negative control experiments. Embryos were considered E0.5 at noon on the day when a plug was discovered. Primers used for genotyping are shown in Table S1. For each experiment, three or more animals from two or more litters were examined. The Animal Care and Use Committee of Stanford University School of Medicine approved all protocols.

### Histology

Cochleae were microdissected in cold Hank's Balanced Salt Solution (HBSS) and prepared as wholemounts or cryosections. Wholemounts were mounted on a coverslip with 0.5 µl of Cell-Tak and all samples were fixed with 4% paraformaldehyde in phosphate-buffered saline (PBS, pH 7.4) for 1 h at room temperature for wholemounts or overnight at 4°C for cryosections. For cryosections, otic capsules were first rinsed with PBS, then treated with a sucrose gradient (10-30% in PBS). The cryosection samples were embedded in 100% optimal cutting temperature (O.C.T.) compound overnight at 4°C, then frozen on dry ice and oriented with the round window facing down. Embedded cryosection samples were stored at −80°C. A Sakura cryostat at −20°C was used to cut 10 µm sections, which were mounted on either SuperFrost Plus slides (Thermo Fisher Scientific) or Gold Seal Ultrastick adhesion slides (Electron Microscopy Sciences). Slides were stored at −20°C until use.

### RNAscope *in situ* hybridization

RNA transcripts were detected using ACDBio RNAscope technology (Red kit V2.5 HD, 322350) on cryosection samples. Manufacturer instructions were followed, except that tissue was boiled for 90 s and Protease Plus was diluted 1:3 in RNAase-free PBS. The following probes were used: DapB (310043), Polr2a (312471), Axin2 (400331) and Lgr5 (312171). BaseScope technology (BaseScope Red, 322971) was used to design probes that specifically detected the exon junction between exon 2 and 4 in the *Ctnnb1^fl(ex3)/+^* mice (Harada et al., 1999). The same modifications to the manufacturer's instructions were used as for RNAscope. Probes included DapB 1zz (701021), Ppib 1zz (701081) and Ctnnb1ex2/4 1zz (Cat: 707651).

### Immunohistochemistry

Both wholemounts and sections were blocked with primary antiserum for at least 1 h. Samples were then incubated overnight at 4°C with primary antibodies. The primary antibodies used were: Atoh1 (rabbit, 1:1000, 21215-1-AP, Proteintech), β-catenin (mouse IgG1, 1:1000, 610153, BD Biosciences), Pou3f4 (rabbit, 1:500, gift of B. Crenshaw, University of Pennsylvania, USA), cyclin D1 (rabbit, 1:250, RM-2113-S0, Thermo Fisher Scientific), E-cadherin (rat IgG1, 1:1000, 14-3249-80, Affymetrix), Epcam (rat IgG1, 1:100-1:200, 118202, BioLegend), Jag1 (goat, 1:500, sc-6011, Santa Cruz Biotechnology), keratin 8 (rat IgG1, 1:200, TromaI-S, Developmental Studies Hybridoma Bank), Ki67 (rabbit, 1:500 or 1:250, ab16667, Abcam), LEF1 (rabbit, 1:200, 2230, Cell Signaling Technology), Myo7a (rabbit, 1:1000, 25-6790, Proteus Bioscience), Prox1 (goat, 1:400, AF2727, R&D Systems), Sox2 [goat, 1:200, sc-17320(Y-17), Santa Cruz Biotechnology], Sox9 (rabbit, 1:1000, AB5535, Millipore), vimentin (rabbit, 1:100, 5741, Cell Signaling Technology), Zeb1 (rabbit, 1:250, 21544-1-AP, Proteintech), and ZO1 (mouse IgG1, 1:500, 33-9100, Thermo Fisher Scientific).

Following incubation with primary antibodies, samples were washed with PBS and incubated with Alexa Fluor 488 and 647 secondary antibodies (A21206, A11055, A31573, A21447, A21247, A21208, A31571, Invitrogen) at a dilution of 1:500 and DAPI (1:10,000) at room temperature for over 2 h. Phalloidin Alexa488 or Alexa647 (1:50-1:2000, A12379 or A22287, Invitrogen) was applied during secondary antibody incubation to label F-actin. EdU detection was performed using the 647 Click-IT kit (C10340, Invitrogen) before proceeding with immunohistochemistry as per the manufacturer's instructions. After rinsing with PBS, whole-mount samples were mounted with Prolong Gold (ProLong^®^ Gold Antifade Reagent, P36930, Thermo Fisher Scientific) and section samples were mounted with DAKO (IVD DAKO mounting medium, S3023, DAKO), Fluorosave (Fluorosave™ reagent, EMD Millipore, 325789) or Prolong Gold.

### Imaging

Samples were imaged using a Zeiss LSM700 or 880 confocal microscope and Zen software or a Zeiss LSM5 Exciter AxioImager M1 epifluorescent microscope (Zeiss). Images were captured in the apex, middle and base turns of the cochleae. From sections, images of the modiolus, spiral ligament and interscalar septum were taken. ImageJ software (National Institutes of Health) was used to analyze images and for cell counting.

### Quantification of foci cells and protein expression

Foci were designated when clusters of at least three cells were found. Other qualitative features used to distinguish cell clusters included nuclei morphology (small, oblong-shaped and densely packed), tdTomato signal (intense) and organization of cells (disruption, especially in the organ of Corti). Foci counts were assessed per region in wholemounts or mid-modiolar cochlear sections. At least three sections were analyzed per animal. Within individual foci, frequency of markers expressed was counted in image stacks of wholemounts or sections.

To assess expression of protein markers in the periotic mesenchyme in control animals, 20-40 DAPI^+^ cells were randomly selected per image. At least three animals from different litters were used (encompassing modiolus, spiral ligament and interscalar septum).

To measure the expression levels of Pou3f4, Sox9 and β-catenin, images of cochlear sections from control and experimental groups were first captured using identical microscope settings. Fluorescence intensity was then measured in a standard region of interest (ROI, 1.26-1.98 µm^2^) centered over the nuclei using ImageJ. The averaged background level was subtracted from the mean intensity of each cell and the average adjusted mean intensity of each animal was calculated.

The intensity of E-cadherin or Jag1 protein expression was measured in cochlear wholemounts. Using the segmented line tool in ImageJ to delineate the cell membrane, the mean intensity of E-cadherin expression was measured at the supporting cell level and corrected for background measurements.

### Statistical analyses

Statistical analysis was conducted using two-tailed unpaired Student's *t*-test, one-way ANOVA with post-hoc Tukey's tests or Chi-squared test in Excel (Microsoft) and GraphPad Prism (GraphPad Software). Statistical significance was set at *P*<0.05. Data from animals within a single litter was combined and treated as a single sample because tamoxifen was administered prenatally as a single dose to each litter.

## Supplementary Material

Supplementary information

Reviewer comments
