# Supplementary material for: Opposing effects of Wnt/β-catenin signaling on epithelial and mesenchymal cell fate in the developing cochlea
Source: Development. 2021 Jun 1;148(11):dev199091. doi: 10.1242/dev.199091 (PMC8217710; doi:10.1242/dev.199091)
Supplement: Supplementary information [file develop-148-199091-s1.pdf]

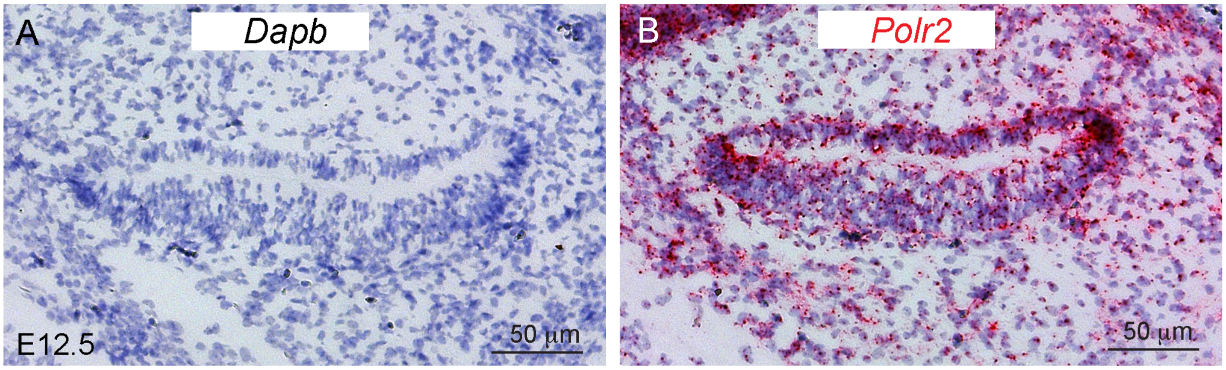

<u>Figure S1. RNAscope *in situ* hybridization</u>

Sections from E12.5 wildtype cochlea. *In situ* hybridization staining using the probe *Dapb* (A) as negative controls and the positive and *Polr2* (B) as positive controls.

Figure S2

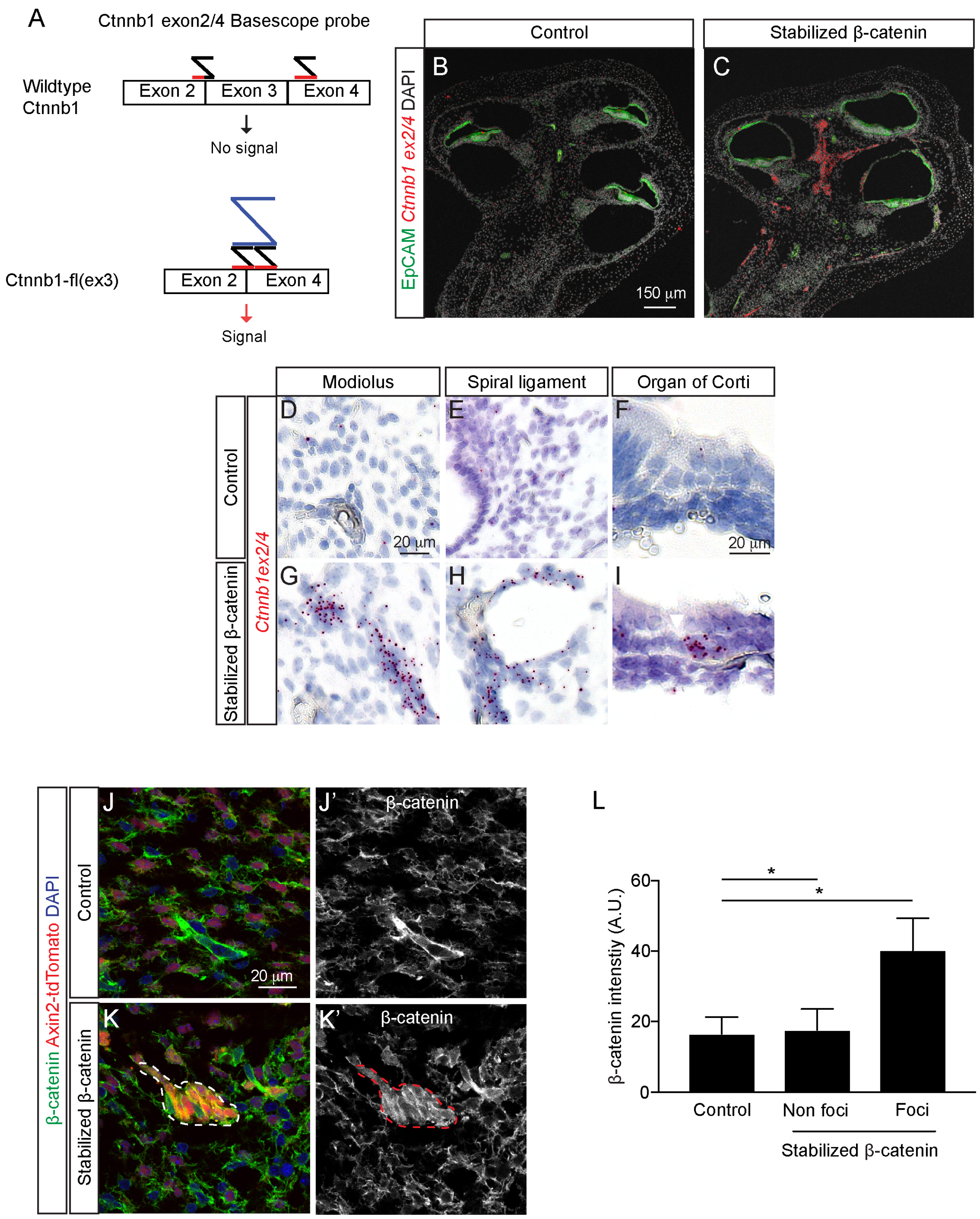

Development • Supplementary information

<u>Figure S2. Validating β-catenin stabilization *in vivo*</u>

A) To detect recombined cells with excised exon 3 of *Ctnnb1* in Axin2-Ctnnb1 cochlea, we used Basescope *in situ* hybridization technology to detect the presence of exon 2/4 junction. The Basescope probe was designed using a single Z-pair, one of which spans exon 2 and 3, and the other located in exon 4. In the absence of exon 3, the proximity of the Z-pair generate a signal. B) Sections from E19.5 control Axin2-tdtomato mice showing no to minimal expression of *Ctnnb1 ex2/4* in the cochlear duct or periotic

mesenchyme. EpCAM (green) robustly labels the cochlear duct, and occasionally blood vessels in the periotic mesenchyme. C) In the Axin2-Ctnnb1 cochlea, foci in the periotic mesenchyme highly expressed *Ctnnb1 ex2/4*. D-F) High magnification, light microscopy images showing almost no expression of *Ctnnb1 ex2/4* in the modiolus, spiral ligament, and organ of Corti from control cochlea. G-I) By contrast, robust expression of *Ctnnb1 ex2/4* was detected in foci from these regions from Axin2-Ctnnb1 cochlea. J-K) Immunostaining of β-catenin in sections of E19.5 control (Axin2-tdTomato) and beta- catenin-stabilized (Axin2-tdTomato-Ctnnb1) cochlea. Foci from beta-catenin-stabilized cochlea expressed higher levels of nuclear β-catenin. L) Quantification of nuclear β-

catenin signal showing significantly higher levels of nuclear β-catenin in foci cells than control cells. Foci cells were identified by their characteristic DAPI$^+$ slender nuclei and higher expression levels of β-catenin. Control cells included non-foci cells within the same image from Axin2-tdTomato-Ctnnb1 cochlea, and cells from control Axin2- tdTomato cochlea. Data shown as mean±s.d., n=150-200 nuclei from 3 litters. One-way ANOVA with Tukey's multiple comparisons test. *$p<0.05$.

Development • Supplementary information

Figure S3

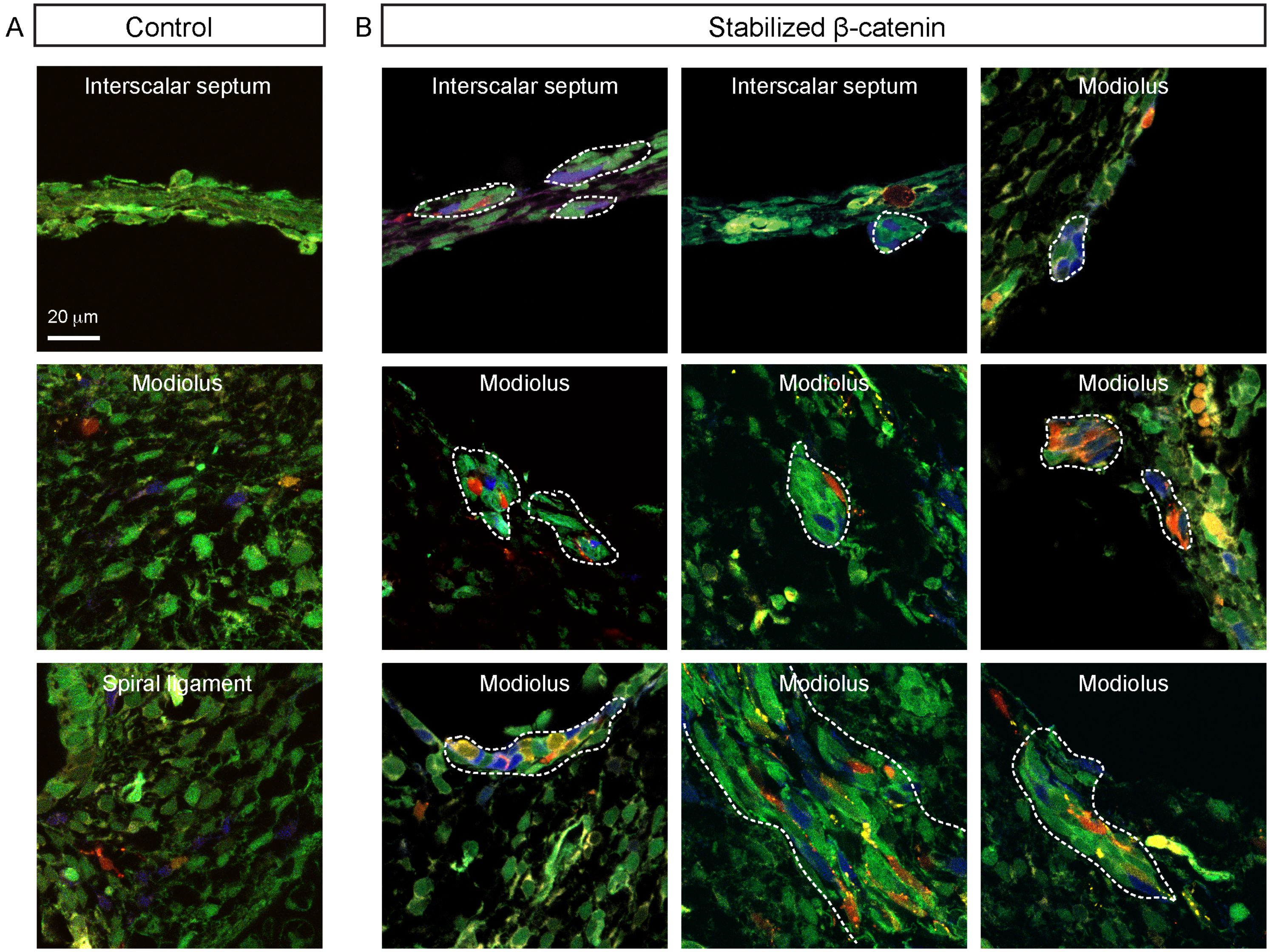

Figure S3 Clonal analyses of β-catenin stabilized foci cells in the periotic mesenchyme

A) Representative images of sections from control (Axin2-Rainbow) cochlea showing the absence of foci and sparse (6.25±3%) Rainbow-traced cells (mCerulean, mOrange, or mCherry) in the interscalar septum, modiolus and spiral ligament. B) In the β-catenin stabilized (Axin2-Rainbow-Ctnnb1) cochlea, almost all foci found in interscalar septum and modiolus were multi-colored, suggesting foci originated from multiple Axin2^{+} cells.

Development • Supplementary information

Figure S4

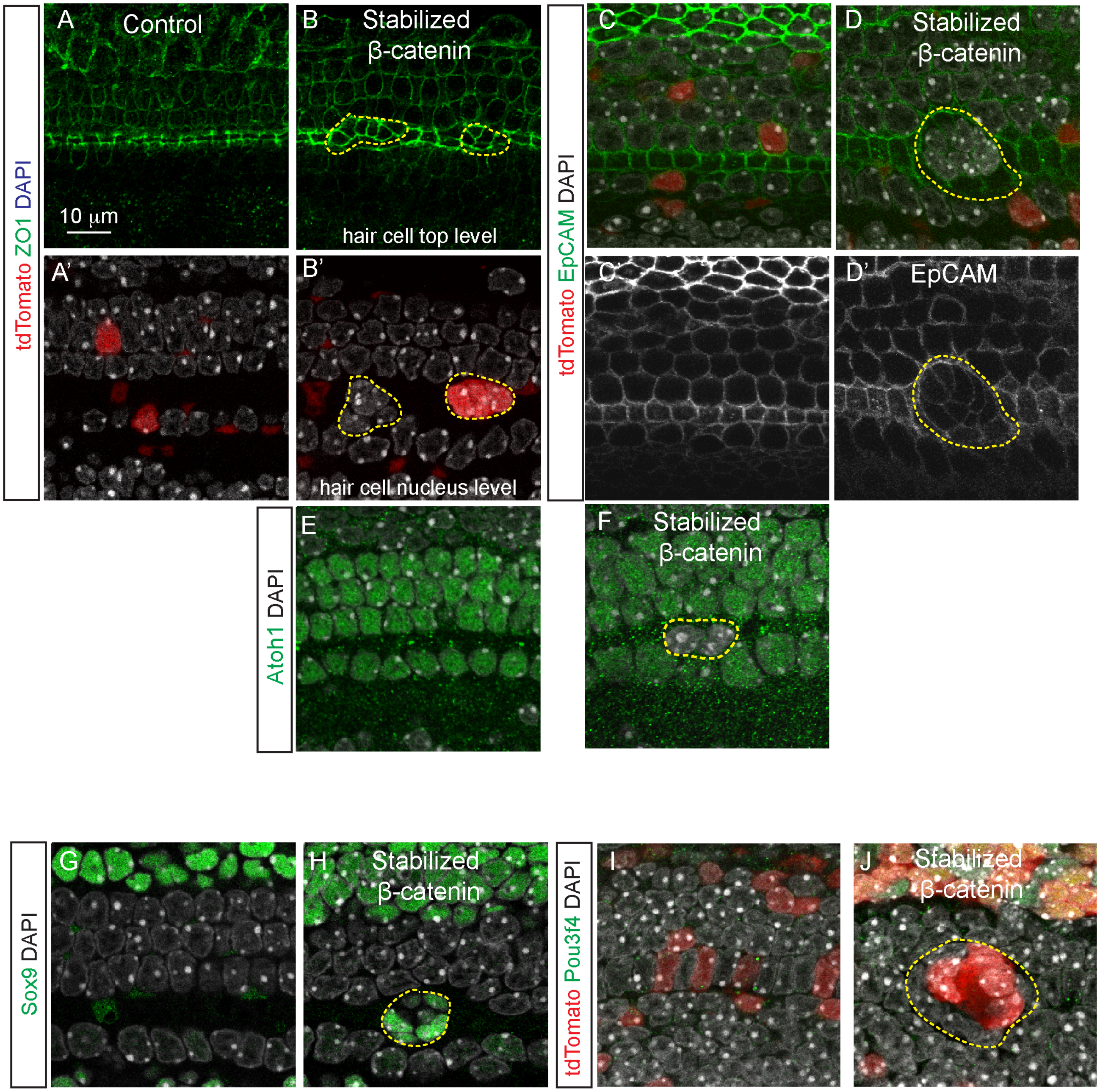

tdTomato ZO1 DAPI
A
Control
10 µm
B
Stabilized β-catenin
hair cell top level
A'
B'
hair cell nucleus level
tdTomato EpCAM DAPI
C
D
Stabilized β-catenin
C'
D'
EpCAM
Atoh1 DAPI
E
F
Stabilized β-catenin
Sox9 DAPI
G
H
Stabilized β-catenin
tdTomato Pou3f4 DAPI
I
J
Stabilized β-catenin

Figure S4 Characteristics of Axin2-derived β-catenin stabilized foci cells in the organ of Corti

A-B) ZO1 expression in organ of Corti cells in E19.5 control (Axin2-tdTomato) and β- catenin stabilized (Axin2-tdTomato-Ctnnb1) cochlea. ZO1 expression is limited to the lumenal surface and was present albeit distorted in foci cells (dashed lines) in β-catenin stabilized cochlea. C-D) Normally expressed in control cochlea, EpCAM appeared reduced in foci cells in β-catenin stabilized cochlea. E-F) Atoh1 expression in hair cells in control cochlea. Foci cells in β-catenin stabilized cochlea lacked Atoh1 expression. G-H) Sox9 expression in supporting cells, but not hair cells, in control cochlea. Foci cells in β-catenin stabilized cochlea were Sox9-positive. I-J) Pou3f4 expression is absent in the organ of Corti from both control and β-catenin stabilized cochleae.

Figure S5

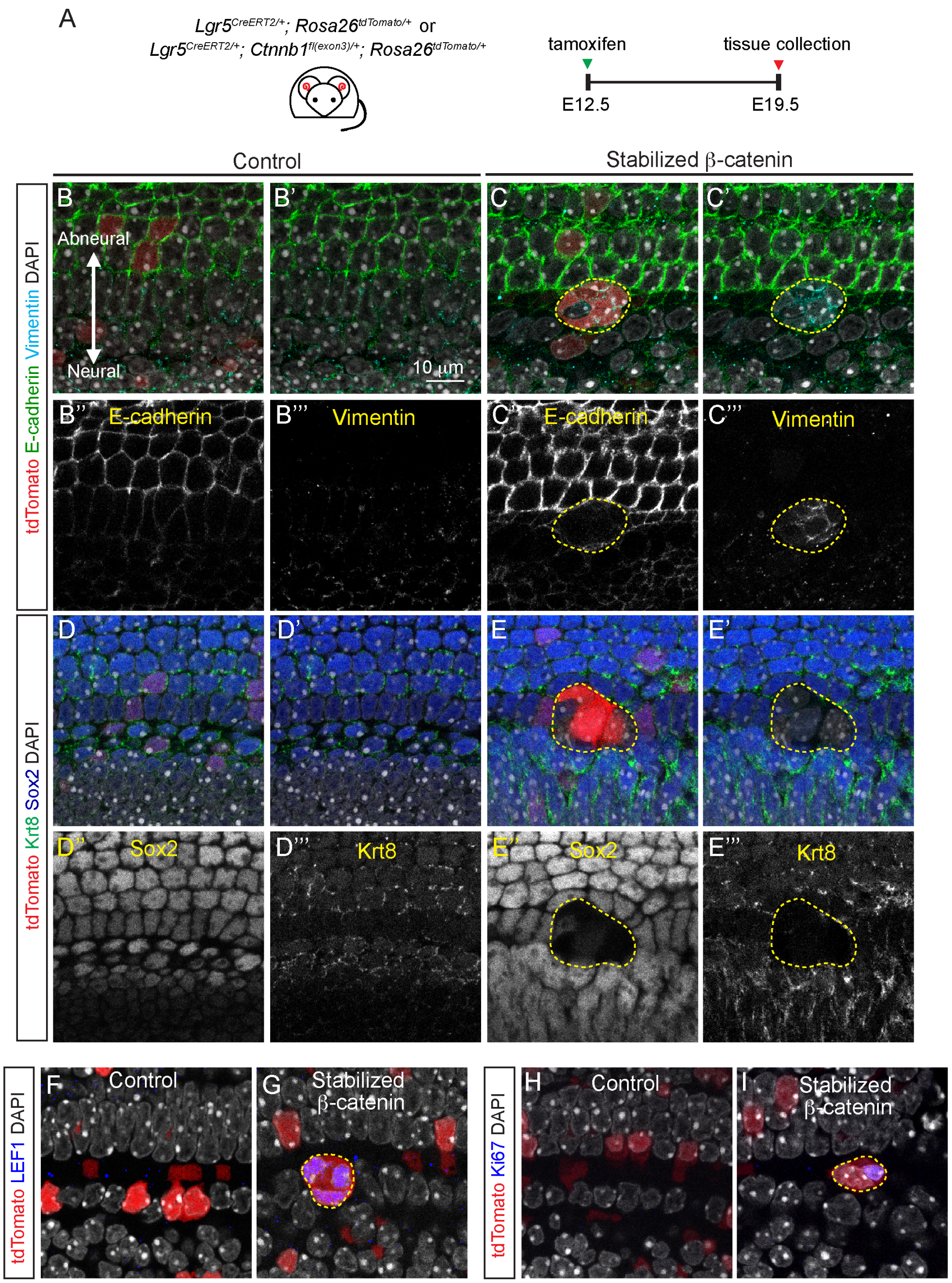
A
Lgr5CreERT2/+; Rosa26tdTomato/+ or
Lgr5CreERT2/+; Ctnnb1fl(exon3)/+; Rosa26tdTomato/+
tamoxifen
tissue collection
E12.5
E19.5
Control
Stabilized β-catenin
tdTomato E-cadherin Vimentin DAPI
B
B'
C
C'
Abneural
Neural
10 μm
B'' E-cadherin
B''' Vimentin
C'' E-cadherin
C''' Vimentin
tdTomato Krt8 Sox2 DAPI
D
D'
E
E'
D'' Sox2
D''' Krt8
E'' Sox2
E''' Krt8
tdTomato LEF1 DAPI
F Control
G Stabilized β-catenin
tdTomato Ki67 DAPI
H Control
I Stabilized β-catenin

Figure S5 Characteristics of Lgr5-derived β-catenin stabilized foci cells in the organ of Corti

A) Experimental scheme to stabilize β-catenin in Lgr5-Ctnnb1 cochlea. B-B''') In control cochlea, E-cadherin was robustly expressed in supporting cells of the organ of Corti, while Vimentin is almost absent. C-C''') With β-catenin stabilization, foci displayed no or reduced E-cadherin expression, but strongly expressed Vimentin. D-D''') Control cochlea with supporting cells expressing Sox2 and keratin 8 (Krt8). E-E''') Few foci in the organ of Corti from Lgr5-Ctnnb1 cochlea showed Krt8 or Sox2 expression. F-G) Absent in control organ of Corti, LEF1 was robustly expressed in Lgr5-Ctnnb1 foci. H) Organ of Corti cells in control cochlea did not express the proliferation marker Ki67. I) Lgr5-Ctnnb1 foci expressed Ki67, indicating active proliferation. n=3 cochlea from 3 mice.

Development • Supplementary information

Figure S6

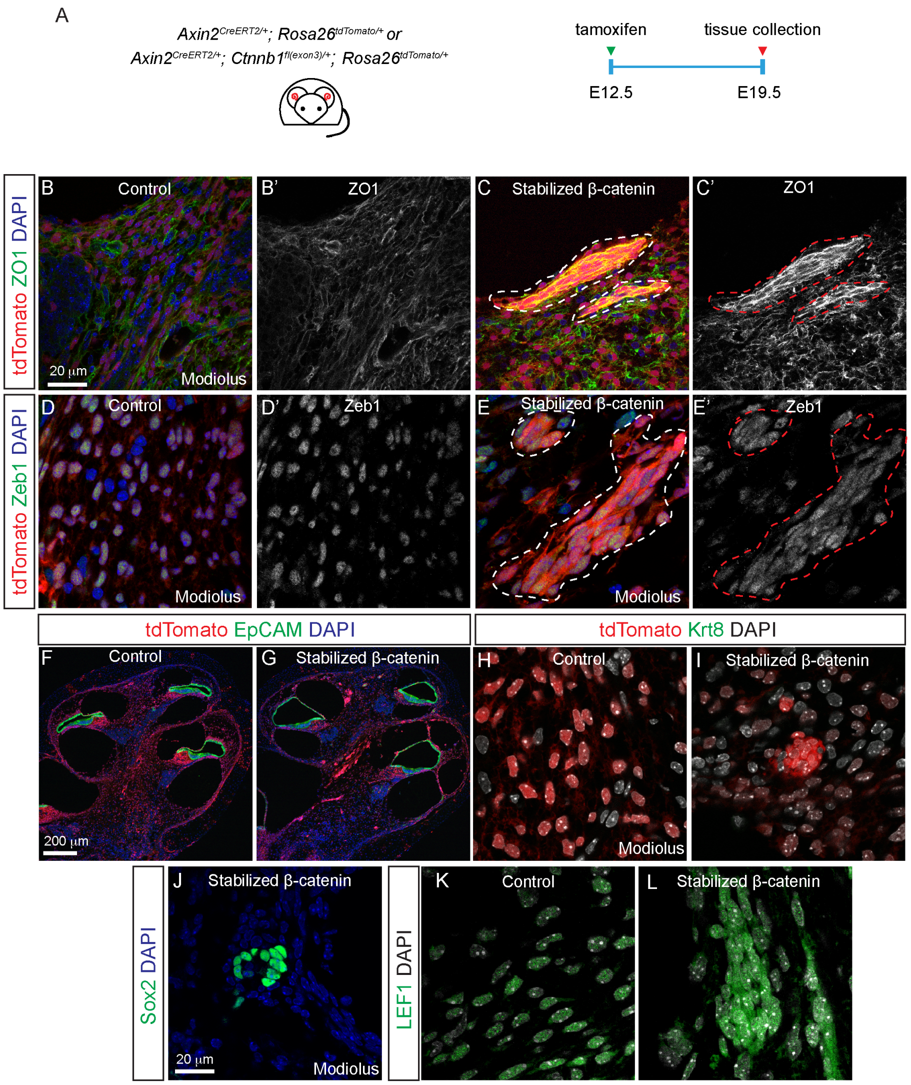

Development • Supplementary information

Figure S6 Characteristics of β-catenin stabilized foci cells in the periotic mesenchyme

A) Experimental scheme depicting control (Axin2-tdTomato) and β-catenin stabilized (Axin2-tdTomato-Ctnnb1) cochleae. B-C) ZO1 expression was low in mesenchymal cells in the modiolus from control cochlea, and was upregulated in foci cells from the β- catenin-stabilized cochleae. D-E) Zeb1 was similarly expressed in both the mesenchymal cells from control cochlea and foci cells from the β-catenin stabilized cochlea. F-G) EpCAM expression in the cochlear duct in both control and β-catenin stabilized cochleae. No ectopic expression in foci cells was observed. H-I) Mesenchymal cells from both the control and β-catenin stabilized cochlea lacked keratin 8 (Krt8) expression. J) Ectopic Sox2 expression in foci in the modiolus of β-catenin stabilized cochlea. L-M) LEF1 expression was similar in mesenchymal cells from both the control and β-catenin stabilized cochlea.

Development • Supplementary information

Table S1. Primers for genotyping and quantitative PCR

Cre recombinase (for Lgr5-EGFP-CreERT2)	Forward	5'-CGATGCAACGAGTGATGAGGT-3'
	Reverse	5'-GCACGTTCACCGGCATCAAC-3'
Rosa26R-tdTomato	WT Forward	5'- AAGGGAGCTGCAGTGGAGTA-3'
	WT Reverse	5'-AAAATCTGTGGGAAGTC-3'
	Mutant Forward	5'-GGCATTAAAGCAGCGTATCC-3'
	Mutant Reverse	5'-CTGTTCCTGTACGGCATGG-3'
Axin2-CreERT2	WT Forward	5'-AAGCTGCGTCGGATACTTGAG-3'
	WT Reverse	5'-AGTCCATCTTCATTCCGCCTA-3'
	Mutant Forward	5'-TGGTAATGCTGCAGTGGCTTG-3'
Catnb-flox (exon3)	Forward	5'-AACTGGCTTTTGGTGTCGGG-3'
	Reverse	5'-TCGGTGGCTTGCTGATTATTTC-3'
Rosa26R-Confetti	Mutant Forward	5'-GAA TTA ATT CCG GTA TAA CTT CG-3'
	WT Forward	5'-AAA GTC GCT CTG AGT TGT TAT-3'
	Reverse	5'-CCA GAT GAC TAC CTA TCC TC-3'
For floxed allele detection		
Ctnnb1 BCAT-F1	WT Forward	5'-GGTAGTGGTCCCTGCCCTTGACAC-3'
Ctnnb1 P85	WT Reverse	5'-CTA AGC TTG GCT GGA CGT AAA CTC-3'
Ctnnb1 GF2	Mutant Forward	5'-GGTAGGTGAAGCTCAGCGCAGAGC-3'
Ctnnb1 AS5	Mutant Reverse	5'-ACGTGTGGCAAGTTCCGCGTCATCC-3'
