# Supplementary material for: Opposing effects of Wnt/β-catenin signaling on epithelial and mesenchymal cell fate in the developing cochlea
Source: Development. 2021 Jun 1;148(11):dev199091. doi: 10.1242/dev.199091 (PMC8217710; doi:10.1242/dev.199091)
Supplement: Reviewer comments [file develop-148-199091_review_history.pdf]

Opposing effects of Wnt/β-catenin signaling on epithelial and mesenchymal cell fate in the developing cochlea

Sara E. Billings, Nina M. Myers, Lee Quiruz and Alan G. Cheng
DOI: 10.1242/dev.199091

Editor: Patrick Tam

Review timeline

Original submission:	30 November 2020
Editorial decision:	18 January 2021
First revision received:	20 April 2021
Accepted:	5 May 2021

Original submission

First decision letter

MS ID#: DEVELOP/2020/199091

MS TITLE: Opposing effects of Wnt/β-catenin signaling on epithelial and mesenchymal cell fate in the developing cochlea

AUTHORS: Alan Cheng, Sara Billings, Nina Myers, and Lee Quiruz

I have now received all the referees' reports on the above manuscript, and have reached a decision. The referees' comments are appended below, or you can access them online: please go to BenchPress and click on the 'Manuscripts with Decisions' queue in the Author Area.

As you will see, the referees express considerable interest in your work, but have raised some concerns and recommend a revision of your manuscript before we can consider publication. If you are able to revise the manuscript along the lines suggested, which may involve further experiments, I will be happy receive a revised version of the manuscript. Your revised paper will be re-reviewed by one or more of the original referees, and acceptance of your manuscript will depend on your addressing satisfactorily the reviewers' major concerns.

We are aware that you may be experiencing disruption to the normal running of your lab that make experimental revisions challenging. If it would be helpful, we encourage you to contact us to discuss your revision in greater detail. Please send us a point-by-point response indicating where you are able to address concerns raised (either experimentally or by changes to the text) and where you will not be able to do so within the normal timeframe of a revision. We will then provide further guidance. Please also note that we are happy to extend revision timeframes as necessary.

Please attend to all of the reviewers' comments and ensure that you clearly highlight all changes made in the revised manuscript. Please avoid using 'Tracked changes' in Word files as these are lost in PDF conversion. I should be grateful if you would also provide a point-by-point response detailing how you have dealt with the points raised by the reviewers in the 'Response to Reviewers' box. If you do not agree with any of their criticisms or suggestions please explain clearly why this is so.

© 2021. Published by The Company of Biologists under the terms of the Creative Commons Attribution License (https://creativecommons.org/licenses/by/4.0/).

Reviewer 1

Advance summary and potential significance to field

Canonical Wnt signaling is known to be required for multiple aspects of proper cochlea development.
Previous work had demonstrated Wnt signaling in the cochlear duct and the surrounding periotic mesoderm (POM) to promote proliferation in both of these compartments. Here, Billings and colleagues provide evidence that Wnt activation promotes proliferation and partial EMT in the cochlear duct while reducing proliferation and inducing partial MET in the POM. The authors use two inducible Cre-driver lines to express a constitutively active form of beta-catenin in Wnt-responsive cells in both the OC and POM. In both compartments, Cre+ cells form foci of closely aggregated cells. IF staining for multiple indicators of cell proliferation point to a local increase in proliferation in OC foci and a decrease in proliferation in POM foci relative to surrounding tissues and cochlear tissues of control mice. Lineage tracing of foci cells using two different multicolor Cre reporter lines demonstrates that OC foci are likely formed by proliferation of individual founder cells, while POM foci are likely formed by aggregation of multiple clones. Finally, the authors perform IF for multiple traditional markers of epithelial cells and mesenchymal cells and find that epithelial markers and markers of cell differentiation are significantly reduced in OC foci, while mesenchymal markers show increased expression. In POM foci, mesenchymal markers are decreased while epithelial markers are increased. beta-catenin activation appears to push the epithelial cells of the OC towards a more mesenchymal state, and mesenchymal POM cells towards an epithelial state, resulting in cells that fall in the intermediate on the epithelial-to-mesenchymal spectrum.

This work demonstrates opposing roles for canonical Wnt signaling in the cochlear epithelium and the surrounding mesenchyme in late embryonic development and highlights the extremely context-dependent nature of the effects of beta-catenin activation. The authors are thorough in their approach: they use multiple mouse models for fate mapping OC foci cells, multiple mouse models for determining the clonality of foci, and examine multiple markers of proliferation and many markers of epithelial and mesenchymal cell identity. The authors provide quantification where appropriate in many instances.

Comments for the author

A few minor points:
1. In some cases statistical analysis does not correct for multiple testing, and only students t-tests are used.
When more than two groups are being compared, it would be better to use ANOVA and post-hoc comparisons (e.g. fig 6 L,O). I do not think re-analysis will alter any conclusions but better to use the appropriate analysis.
2. The white box in Fig 2K is displaced.
3. The groups in Fig 6 L,O appear to be mis-lableled, that is, foci have lower expression than non-foci and controls as described in the text and demonstrated in J,K,M,N, while the graph indicates that controls have the lower expression.

Reviewer 2

Advance summary and potential significance to field

This study by Billings et al., uncovers that aberrant Wnt/beta-catenin signaling has strikingly different effects on epithelial and mesenchymal cells in the developing cochlea. The authors find that over-activate Wnt/beta-catenin signaling in Axin2+ cells at stage E12.5 (Axin2-CreER/+; Ctnnb1fl(ex3)/+, tamoxifen at E12.5) leads to the formation of ectopic cell clusters (foci) within the periotic mesenchyme (POM) and the otic epithelium.
The authors then make the interesting discovery that while the otic foci are the product of ectopic proliferation and have diminished expression of epithelial and differentiation markers and show ectopic mesenchymal marker expression, POM foci are the product of aberrant cell aggregation characterized by diminished cell proliferation, a lack of mesenchymal marker expression and ectopic epithelial marker expression.

© 2021. Published by The Company of Biologists under the terms of the Creative Commons Attribution License (https://creativecommons.org/licenses/by/4.0/).

Taken together these findings indicate that increased Wnt/beta-catenin signaling triggers cell fate transition between epithelial and mesenchymal cell states in the developing murine cochlea.

Comments for the author

This is an exceptional well designed and executed study and I have only few suggestions for improvement.

Major:
The authors find that Wnt/beta-catenin overactivation at stage E12.5 in Axin2+ cells results in cell clusters (foci) within the cochlear sensory epithelium with mesenchymal cell-like characteristics. The authors conclude that Wnt/beta-catenin overactivation triggered epithelial-mesenchymal transition (EMT). This is a reasonable interpretation of the data, however given that at stage E12.5 Axin2 is mainly expressed in periotic mesenchymal cells, it is similarly possible that the observed cell clusters are the product of disrupted or incomplete mesenchymal-to-epithelial transition of Axin2+ mesenchymal cells. To confirm or refute such alternative explanation the authors should analyze mesenchymal and epithelial marker expression in otic foci produced by activation of aberrant Wnt/beta-catenin signaling in Lgr5+ cells cochlear epithelial cells (Lgr5-CreER/+ Ctnnb1fl(ex3)/+, TM E12.5), shown in Fig.2).

Minor:
1) The provided schematic in Fig.1 could be improved by arrows or color to more clearly mark the named anatomical structures.

2) Consistent with previous reports, the authors find that stage E12.5 Lgr5+ cells give rise to hair cells and supporting cells (organ of Corti). The provided image reveals that Lgr5-tdTomato+ cells are also found outside the organ of Corti (Fig. 1 H'). Thus, a quantification of the % Lgr5-tdTomato+ found outside the organ of Corti e.g. stria vascularis would be informative.

3) The authors show that overactivation of Wnt/beta-catenin in stage E12.5 Lgr5+ cells (Lgr5-CreER/+ Ctnnb1fl(ex3)/+ R26-tdTomato mice) results in cell clusters (foci) in the pillar cell region. Similar foci have been reported after overactivation of Wnt/beta-catenin signaling in perinatal supporting cells, with some of these foci containing ectopic hair cells. It would be of great interest to learn whether Lgr5-derived cell clusters produced by embryonic overactivation of Wnt/beta-catenin signaling contain ectopic hair cells, or whether these cell cluster show diminished epithelial/supporting cell characteristics (see also major critique).

4) The authors should comment on why stage E18.5 was used to analyze the effects of beta-catenin stabilization on mesenchymal and epithelial cell proliferation (Fig.3).

5) The authors state that ‘there was no EdU or Ki67 labeling in organ of Corti of control (Axin2-CreER/+ R26-tdTomato) mice, consistent with previous reports of mitotic quiescence by E19.5'; however no quantitative data is provided.

Given that these experiments were conducted with genetically modified mice (e.g. Axin2 haploinsufficient). I would recommend to include a quantification of % EdU+ and/or % Ki67+ organ of Corti cells (even if 0%).

6) The authors should include the official gene symbols/names for all the genes/gene products named in the study (e.g. Brn4 (Pou3f4)).

7) I recommend to remove the word 'classic' in 'classic epithelial marker (page 13, line 16).

8) The authors state "...In contrast to these studies, we observed a downregulation or loss of markers of epithelial cells or supporting cells after ß-catenin stabilization, increased proliferation and expression of some mesenchymal markers, suggestive of dedifferentiation and EMT".

© 2021. Published by The Company of Biologists under the terms of the Creative Commons Attribution License (https://creativecommons.org/licenses/by/4.0/).

I would recommend removing ..'increase proliferation' as increase proliferation was also noted in aforementioned studies.

First revision

Author response to reviewers' comments

Point-by-point responses

Reviewer 1 Advance summary and potential significance to field
Canonical Wnt signaling is known to be required for multiple aspects of proper cochlea development. Previous work had demonstrated Wnt signaling in the cochlear duct and the surrounding periotic mesoderm (POM) to promote proliferation in both of these compartments. Here, Billings and colleagues provide evidence that Wnt activation promotes proliferation and partial EMT in the cochlear duct while reducing proliferation and inducing partial MET in the POM. The authors use two inducible Cre-driver lines to express a constitutively active form of beta-catenin in Wnt-responsive cells in both the OC and POM. In both compartments, Cre+ cells form foci of closely aggregated cells. IF staining for multiple indicators of cell proliferation point to a local increase in proliferation in OC foci and a decrease in proliferation in POM foci relative to surrounding tissues and cochlear tissues of control mice. Lineage tracing of foci cells using two different multicolor Cre reporter lines demonstrates that OC foci are likely formed by proliferation of individual founder cells, while POM foci are likely formed by aggregation of multiple clones. Finally, the authors perform IF for multiple traditional markers of epithelial cells and mesenchymal cells and find that epithelial markers and markers of cell differentiation are significantly reduced in OC foci, while mesenchymal markers show increased expression. In POM foci, mesenchymal markers are decreased while epithelial markers are increased. beta-catenin activation appears to push the epithelial cells of the OC towards a more mesenchymal state, and mesenchymal POM cells towards an epithelial state, resulting in cells that fall in the intermediate on the epithelial-to-mesenchymal spectrum.

This work demonstrates opposing roles for canonical Wnt signaling in the cochlear epithelium and the surrounding mesenchyme in late embryonic development and highlights the extremely context-dependent nature of the effects of beta-catenin activation. The authors are thorough in their approach: they use multiple mouse models for fate mapping OC foci cells, multiple mouse models for determining the clonality
of foci, and examine multiple markers of proliferation and many markers of epithelial and mesenchymal cell identity. The authors provide quantification where appropriate in many instances.

Thank you for your kind words.

Reviewer 1 Comments for the author
A few minor points:

1. In some cases statistical analysis does not correct for multiple testing, and only students t-tests are used. When more than two groups are being compared, it would be better to use ANOVA and post-hoc comparisons (e.g. fig 6 L,O). I do not think re-analysis will alter any conclusions but better to use the appropriate analysis.

We have reanalyzed the data using one-way ANOVA with Tukey's multiple comparisons test. While the significance of most comparisons did not change, a few did and these changes have been made in the results and figure legends describing figures 3, 4, 5, 6, s2.

2. The white box in Fig 2K is displaced.

This has been corrected.

© 2021. Published by The Company of Biologists under the terms of the Creative Commons Attribution License (https://creativecommons.org/licenses/by/4.0/).

3. The groups in Fig 6 L,O appear to be mis-lableled, that is, foci have lower expression than non-foci and controls as described in the text and demonstrated in J,K,M,N, while the graph indicates that controls have the lower expression.

This has been corrected.

Reviewer 2 Advance summary and potential significance to field
This study by Billings et al., uncovers that aberrant Wnt/beta-catenin signaling has strikingly different effects on epithelial and mesenchymal cells in the developing cochlea. The authors find that over-activate Wnt/beta-catenin signaling in Axin2+ cells at stage E12.5 (Axin2-CreER/+;Ctnnb1fl(ex3)/+, tamoxifen at E12.5) leads to the formation of ectopic cell clusters (foci) within the periotic mesenchyme (POM) and the otic epithelium. The authors then make the interesting discovery that while the otic foci are the product of ectopic proliferation and have diminished expression of epithelial and differentiation markers and show ectopic mesenchymal marker expression, POM foci are the product of aberrant cell aggregation, characterized by diminished cell proliferation, a lack of mesenchymal marker expression and ectopic epithelial marker expression. Taken together these findings indicate that increased Wnt/beta-catenin signaling triggers cell fate transition between epithelial and mesenchymal cell states in the developing murine cochlea.

Reviewer 2 Comments for the author
This is an exceptional well designed and executed study and I have only few suggestions for improvement.

Thank you for your kind words.

Major:
The authors find that Wnt/beta-catenin overactivation at stage E12.5 in Axin2+ cells results in cell clusters (foci) within the cochlear sensory epithelium with mesenchymal cell-like characteristics. The authors conclude that Wnt/beta-catenin overactivation triggered epithelial-mesenchymal transition (EMT). This is a reasonable interpretation of the data, however, given that at stage E12.5 Axin2 is mainly expressed in periotic mesenchymal cells, it is similarly possible that the observed cell clusters are the product of disrupted or incomplete mesenchymal-to-epithelial transition of Axin2+ mesenchymal cells. To confirm or refute such alternative explanation, the authors should analyze mesenchymal and epithelial marker expression in otic foci produced by activation of aberrant Wnt/beta-catenin signaling in Lgr5+ cells cochlear epithelial cells (Lgr5-CreER/+ Ctnnb1fl(ex3)/+, TM E12.5), shown in Fig.2).

Thank you for this suggestion. We have examined Lgr5-CreERT2; Ctnnb1-fl/(ex3) cochlea and found that foci formed similarly have diminished expression of epithelial markers (E-cadherin, Sox2, Keratin 8), increased expression of proliferation (Ki67) and mesenchymal markers (Vimentin and LEF1). These results therefore corroborated with those in Axin2-CreERT2; Ctnnb1-fl/(ex3) cochlea. Since Lgr5-traced were exclusively epithelial in origin, these results support our conclusion the beta-catenin stabilization led to an epithelial-mesenchymal transition. This data can be found in the new Figure S5 and described in the results (p.14-15).

Minor:
1) The provided schematic in Fig.1 could be improved by arrows or color to more clearly mark the named anatomical structures.

We have added additional reference marks and colors to more clearly label this figure.

2) Consistent with previous reports, the authors find that stage E12.5 Lgr5+ cells give rise to hair cells and supporting cells (organ of Corti). The provided image reveals that Lgr5-tdTomato+ cells are also found outside the organ of Corti (Fig. 1 H'). Thus, a quantification of the % Lgr5-tdTomato+ found outside the organ of Corti e.g. stria vascularis would be informative.

The tracing of Lgr5+ cells is consistent with the in situ pattern at E12.5, which extends across the entire floor of the cochlear duct. Sparse labeling was expected given the low tamoxifen dose

© 2021. Published by The Company of Biologists under the terms of the Creative Commons Attribution License (https://creativecommons.org/licenses/by/4.0/).

employed. We have quantified Lgr5-tdTomato cells outside of the organ of Corti and added this to the results section (p.8, L1-4).

3) The authors show that overactivation of Wnt/beta-catenin in stage E12.5 Lgr5+ cells (Lgr5-CreER/+ Ctnnb1fl(ex3)/+ R26-tdTomato mice) results in cell clusters (foci) in the pillar cell region. Similar foci have been reported after overactivation of Wnt/beta-catenin signaling in perinatal supporting cells, with some of these foci containing ectopic hair cells. It would be of great interest to learn whether Lgr5-derived cell clusters produced by embryonic overactivation of Wnt/beta-catenin signaling contain ectopic hair
cells, or whether these cell cluster show diminished epithelial/supporting cell characteristics (see also major critique).

We agree that this would be potentially interesting, given that others have shown that postnatal supporting cells can acquire a hair cell fate after beta-catenin overexpression. Unfortunately, almost all of the embryos with beta-catenin overexpression were unable to be born in the postnatal period because of dystocia, particularly for Axin2-CreER/+ Ctnnb1fl(ex3)/+ mice. We have only been able to examine the cochlea of one surviving animal (Lgr5-CreER/+ Ctnnb1fl(ex3)/+ R26-tdTomato) and found that many foci cells were similarly undifferentiated with rare cells expressing Myosin7a. While this postnatal data is consistent with the embryonic data, we have decided not to include it because this experiment could not be repeated.
To address this point, we have added the following to the results section:
(p.14 L11-13) "Analysis of the fate of foci cells at later ages was precluded by tamoxifen-induced dystocia and perinatal lethality of the mutant animals."

4) The authors should comment on why stage E18.5 was used to analyze the effects of beta-catenin stabilization on mesenchymal and epithelial cell proliferation (Fig.3).

EdU was administered at E18.5 prior to tissue harvest at E19.5 to simply label proliferative cells. E19.5 was used because Axin2; Ctnnb1 animals do not survive after birth. We also presumed that this allowed time for beta-catenin to induce phenotypic changes such as proliferation in Wnt-responsive cells at a stage when cells are relatively more differentiated than earlier time points.

5) The authors state that 'there was no EdU or Ki67 labeling in organ of Corti of control (Axin2-CreER/+ R26-tdTomato) mice, consistent with previous reports of mitotic quiescence by E19.5'; however no quantitative data is provided. Given that these experiments were conducted with genetically modified mice (e.g. Axin2 haploinsufficient). I would recommend to include a quantification of % EdU+ and/or % Ki67+ organ of Corti cells (even if 0%).

We have included this data in the results section (p. 11, L3-5).

6) The authors should include the official gene symbols/names for all the genes/gene products named in the study (e.g. Brn4 (Pou3f4)).

We have now included this throughout the text and figures.

7) I recommend to remove the word 'classic' in 'classic epithelial marker (page 13, line 16).

This has been removed.

8) The authors state "...In contrast to these studies, we observed a downregulation or loss of markers of epithelial cells or supporting cells after ß-catenin stabilization, increased proliferation and expression of some mesenchymal markers, suggestive of dedifferentiation and EMT".
I would recommend removing ..'increase proliferation' as increase proliferation was also noted in aforementioned studies.

© 2021. Published by The Company of Biologists under the terms of the Creative Commons Attribution License (https://creativecommons.org/licenses/by/4.0/).

This has been removed.

Second decision letter

MS ID#: DEVELOP/2020/199091

MS TITLE: Opposing effects of Wnt/ß-catenin signaling on epithelial and mesenchymal cell fate in the developing cochlea

AUTHORS: Sara Billings, Nina Myers, Lee Quiruz, and Alan Cheng
ARTICLE TYPE: Research Article

I am happy to tell you that your manuscript has been accepted for publication in Development, pending our standard ethics checks.

Reviewer 2

Advance summary and potential significance to field

The study by Billings et al., uncovers that aberrant Wnt/beta-catenin signaling has strikingly different effects on epithelial and mesenchymal cells in the developing cochlea. The authors find that beta-catenin stabilization leads to the formation of ectopic cell clusters (foci) within the periotic mesenchyme (POM) and the otic epithelium, with distinctly different characteristics. While the otic foci are the product of ectopic proliferation and show ectopic mesenchymal marker expression, POM foci are the product of aberrant cell aggregation, characterized by diminished cell proliferation, and ectopic epithelial marker expression. In summary, the author's findings provide compelling evidence that aberrant Wnt/beta-catenin activation in the developing cochlea alters epithelial and mesenchymal cell characteristics triggering epithelial to mesenchymal and mesenchymal to epithelial cell state transitions.

Comments for the author

The authors addressed all concerns raised by both reviewers satisfactory. I have no further suggestions/comments for improvement.

© 2021. Published by The Company of Biologists under the terms of the Creative Commons Attribution License (https://creativecommons.org/licenses/by/4.0/).
